# Utilizing ion leaching effects for achieving high oxygen-evolving performance on hybrid nanocomposite with self-optimized behaviors

**DOI:** 10.1038/s41467-020-17108-5

**Published:** 2020-07-06

**Authors:** Daqin Guan, Gihun Ryu, Zhiwei Hu, Jing Zhou, Chung-Li Dong, Yu-Cheng Huang, Kaifeng Zhang, Yijun Zhong, Alexander C. Komarek, Ming Zhu, Xinhao Wu, Chih-Wen Pao, Chung-Kai Chang, Hong-Ji Lin, Chien-Te Chen, Wei Zhou, Zongping Shao

**Affiliations:** 1 State Key Laboratory of Materials-Oriented Chemical Engineering, College of Chemical Engineering, Nanjing Tech University, Nanjing, 211800 China; 2 Max-Planck-Institute for Chemical Physics of Solids, Nöthnitzer Str. 40, Dresden, 01187 Germany; 3 Shanghai Institute of Applied Physics, Chinese Academy of Sciences, Shanghai, 201204 China; 4 Department of Physics, Tamkang University, 151 Yingzhuan Rd., New Taipei City, 25137 Taiwan; 5 WA School of Mines: Minerals, Energy and Chemical Engineering (WASM-MECE), Curtin University, Perth, WA 6845 Australia; 6 National Synchrotron Radiation Research Center, 101 Hsin-Ann Road, Hsinchu, 30076 Taiwan

**Keywords:** Electrocatalysis, Electrocatalysis, Nanoparticles

## Abstract

Ion leaching from pure-phase oxygen-evolving electrocatalysts generally exists, leading to the collapse and loss of catalyst crystalline matrix. Here, different from previous design methodologies of pure-phase perovskites, we introduce soluble BaCl_2_ and SrCl_2_ into perovskites through a self-assembly process aimed at simultaneously tuning dual cation/anion leaching effects and optimizing ion match in perovskites to protect the crystalline matrix. As a proof-of-concept, self-assembled hybrid Ba_0.35_Sr_0.65_Co_0.8_Fe_0.2_O_3-*δ*_ (BSCF) nanocomposite (with BaCl_2_ and SrCl_2_) exhibits the low overpotential of 260 mV at 10 mA cm^-2^ in 0.1 M KOH. Multiple *operando* spectroscopic techniques reveal that the pre-leaching of soluble compounds lowers the difference of interfacial ion concentrations and thus endows the host phase in hybrid BSCF with abundant time and space to form stable edge/face-sharing surface structures. These self-optimized crystalline structures show stable lattice oxygen active sites and short reaction pathways between Co–Co/Fe metal active sites to trigger favorable adsorption of OH^−^ species.

## Abstract (web summary)

Water oxidation catalysis may provide the electrons needed for sustainable fuel production, but catalysts often degrade under working conditions. Here, authors introduce soluble species into perovskites to exert positive ion leaching effects for enhancing perovskite stability and activity.

## Introduction

Efficient interconversion of electricity and chemicals by electrochemical processes is key for many sustainable energy systems (e.g., water splitting)^1,2^. As the bottleneck reaction of water–alkali electrolyzers, the sluggish oxygen evolution reaction (OER) hinders the efficiency of hydrogen production, which has long posed one of the biggest challenges in this field^1,2^. Therefore, high-performance, low-cost, and scalable OER electrocatalysts are highly desired. As a promising alternative to precious metal-based (i.e., Ir and Ru) compounds, perovskite oxides (general formula ABO_3_, where A = rare-earth or alkaline-earth metals and B = transition metals), with rich structural (e.g., coordination numbers, space groups) and electronic structural features (e.g., valence, spin states, magnetism), have been brought to the forefront for catalyzing OER in alkaline solutions during the past decade^1,3^. However, to date, the state-of-the-art perovskites^4,5^ still require a large overpotential (*η*) of ≥290 mV to reach a current density of 10 mA cm^−2^ (an important parameter for a solar-to-hydrogen efficiency of 12.3%)^6^ in 0.1 M KOH. Moreover, the structure–activity relationships and mechanisms extracted from ex situ characterizations are being challenged by more and more *operando* studies^7–9^. Optimizing the behaviors of the catalyst surface during OER thus becomes a crucial way to break through the activity bottleneck of perovskites.

Structure–activity relationships and relative design principles derived from ex situ findings are often reported. Many typical perovskite families have been exploited to explore their structure–activity relationships and mechanisms, such as single perovskites ABO_3-*δ*_^10^, single-layer Ruddlesden–Popper (RP) perovskites A_2_BO_4-*δ*_^11^, double perovskites A_2_B_2_O_6-*δ*_^12^, triple perovskites A_3_B_3_O_9-*δ*_^13^, three-layer RP perovskites A_4_B_3_O_10-*δ*_^14^, quadruple perovskites A_4_B_4_O_12-*δ*_^15^, and hexagonal perovskites A_8_B_4_O_15-*δ*_^16^. According to these structure–activity correlations obtained from ex situ characterizations and computations, some activity descriptors are also successfully proposed, including *e*_g_ orbital occupancy^10^, O 2*p* band center^12^, and charge-transfer energy^17^. However, very recent *operando* studies have shown that cations and anions in pure-phase materials tend to leach into the electrolyte during alkaline OER conditions due to the differences of ion concentration between alkaline solutions and pure-phase materials. For examples of cation leaching, Ba^2+^/Sr^2+^ leaching from Ba_0.5_Sr_0.5_Co_0.8_Fe_0.2_O_3-*δ*_ (BSCF5582)^8,18–20^, Li^+^ leaching from Li_2_Co_2_O_4-*δ*_^21^, Co/Fe ion leaching from LaCo_0.8_Fe_0.2_O_3-*δ*_ (LCF)^4^, and Sn^4+^ leaching from SnNiFe^5^ have been reported. In terms of anion leaching, Jiang^22^, Chen^23^ and Zhang^24^ et al. have found Cl^−^ leaching from Co_2_(OH)_3_Cl, F^−^ leaching from F-doped CoOOH and NiFeO_*x*_H_*y*_, respectively. Generally, the ion leaching behaviors in pure-phase materials can trigger favorable OH^−^ adsorption on catalyst surface via the ionic effects to accelerate OER processes^8,18,25^. However, the loss of ions from pure-phase materials would sacrifice the surface crystal configurations to form amorphous low-conductivity surface layers [e.g., oxy(hydroxide) layers]^8,18,20^, leading to the collapse and loss of catalyst crystalline matrix and thus lowering the matrix utilization. Therefore, how to simultaneously benefit from the advantages of ion leaching effects and stabilize perovskite crystalline matrix remains an open question. Compared with pure-phase catalysts, hybrid materials, with much larger physicochemical property degrees of freedom^4,26–28^, can offer tremendous possibilities for us to design promising candidates to exert positive ion leaching effects and protect perovskite crystalline matrix.

It is also worth noting that the original oxidation state in materials plays an important role in the formation of amorphous layers during alkaline OER. For example, low-valence Co ions (Co^2+/3+^) tend to be oxidized to Co^3+/4+^ cations under alkaline OER conditions and this change would lead to the reconstruction, collapse and loss of surface crystalline matrix to form amorphous oxy(hydroxide) layers, as known in the cases of H_2_/Ar reduced LCF^4^ (Co^2+/3+^), Li_2_Co_2_O_4_^21^ (Co^3+^), and BSCF5582^8^ (Co^2+/3+^) fabricated from liquid-state reaction synthesis method. Contrarily, high-valence transition-metal ions (e.g., Co^3+/4+^ couples) could relieve the effects of electro-derived oxidation process for alkaline OER, such as high-valence PrBa_0.5_Sr_0.5_Co_1.5_Fe_0.5_O_5+*δ*_ (Co^3+/4+^)^29^, Ba_4_Sr_4_(Co_0.8_Fe_0.2_)_4_O_15_ (Co^3+/4+^)^16^, and Na_0.67_CoO_2_ (Co^3+/4+^)^30^ with good structural stabilities. To further optimize the pristine ion combination in hybrid materials, we also apply a self-assembly synthesis method.

Here, different from prior design methodologies of pure-phase perovskites, we introduce soluble foreign compounds (namely BaCl_2_ and SrCl_2_) into high-valence perovskite structures via a self-assembly synthesis method for simultaneous achievement of positive anion/cation leaching effects and the protection of perovskite crystalline matrix. Following our design principles, high-valence hybrid Ba_0.35_Sr_0.65_Co_0.8_Fe_0.2_O_3-*δ*_ (BSCF) nanocomposite (Co^3+/4+^) is fabricated through a solid-state reaction method^31^, which exhibits a strong OER performance with a small overpotential of 260 mV required for 10 mA cm^−2^ and a low onset overpotential of 200 mV on glassy carbon electrodes (GCE) in 0.1 M KOH among all cobalt-based perovskites reported so far. This performance is much superior to that of previously reported low-valence BSCF5582 with Co^2+/3+^ ions^18^ and the benchmark IrO_2_ catalyst. To track the evolution of geometric and electronic structures during electrochemical OER processes and elucidate the underlying mechanisms, we apply multiple *operando* techniques, including synchrotron X-ray diffraction (XRD), hard X-ray absorption spectroscopy [XAS of near edge structure (XANES) and extended X-ray absorption fine structure (EXAFS)], and soft XAS. As expected, the pre-leaching of soluble compounds in high-valence hybrid BSCF protects perovskite crystalline matrix by lowering the differences of ion concentration (Ba^2+^ and Sr^2+^ ions) between interfacial liquid and its host phase, along with the creation of stable edge/face-sharing surface structures, leading to a good stability for 100 h under 10 mA cm^−2^_disk_. Compared with high-valence pure-phase BSCF, these self-optimized edge/face-sharing structures on hybrid BSCF show more stable lattice oxygen active sites and shorter reaction pathways between Co and Co/Fe metal active sites, which further improve the OER stability and induce more OH^−^ reactants on the surface to boost the OER kinetics. Furthermore, stable Fe^3+^ ions and slightly enhanced Co–O hybridization in hybrid BSCF can well couple the metal and oxygen active sites. This strategy has also proven to be valid for other typical single, double, and RP perovskites. Our design principle based on *operando* changes of catalysts during electrocatalysis paves a new way to the rational design of hybrid materials for efficient energy conversions.

## Results

### Ion leaching effects during OER

Decades of efforts have been devoted to understanding the OER mechanisms (4OH^−^ → O_2_ + 2H_2_O + 4e^−^) on oxides and the mechanism systems have been successfully established, including conventional adsorbate evolution mechanism (AEM) on active transition-metal sites^10^, lattice oxygen oxidation mechanism (LOM) on active oxygen sites^32^, and cooperative AEM and LOM on metal and oxygen active sites^16,33^, respectively. In all the existing OER mechanisms, absorbed OH*, *O, and *OOH intermediates are always involved. As the most important starting reaction step, the adsorption of OH^−^ species becomes the corner stone for efficient OER processes.

**Fig. 1:**
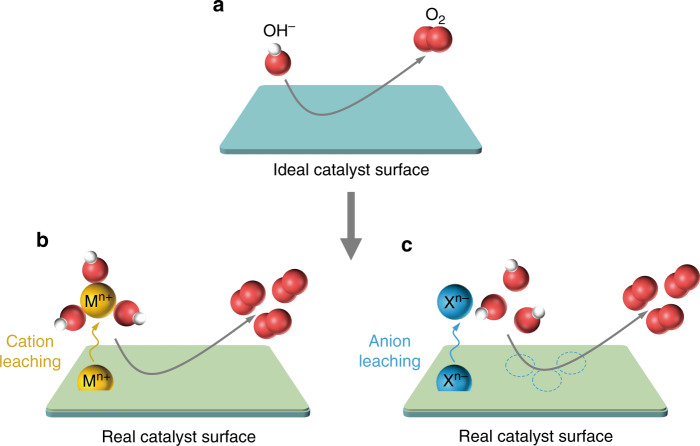
Schematic description of ion leaching effects on catalyst surface during OER in alkaline media. **a** Ideal OER process without ion leaching on material surface. Actual **b** cation leaching and **c** anion leaching behaviors on real catalyst surface during alkaline OER, where the ion leaching effects could contribute to the adsorption of OH^−^ reactants and thus promote OER process.

Based on prior studies^5,8,18,21–24,34,35^, we propose the possible basic models for the variations of electrocatalyst surface triggered by ion leaching effects during OER in alkaline solutions here. Ideally, OH^−^ species on the near surface of catalysts could be adsorbed on active sites and converted into O_2_ molecules as shown in Fig. 1a. However, owing to the differences of interfacial ion concentration and the functions of electro-derived oxidation processes, surface structural oscillation and reconstruction phenomena on catalysts may occur, leading to the leaching of material ions. In terms of cation leaching, the leached cations M^*n*+^ tend to form M^*n*+^-*n*(OH^−^) species on catalyst surface due to the requirement of charge balance, and then induce more OH^−^ reactants in local reaction areas to accelerate OER kinetics^8,18^ (Fig. 1b). As for anion leaching, OH^−^ species would make charge compensation on the unsaturated sites created by leached anions X^*n*−^, and further promote OER processes^22–24^ (Fig. 1c). However, the leaching behaviors of pure-phase materials may destroy and sacrifice the unique active coordination environments of crystalline matrix to form amorphous low-conductivity surface oxy(hydroxide) layers, which could lower the matrix utilization^8,18,20^.

### Self-assembly synthesis and ex situ characterizations

**Fig. 2:**
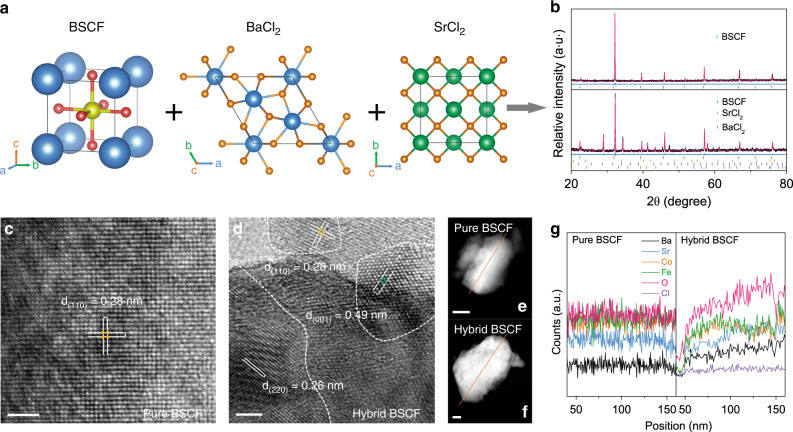
Synthesis and ex situ characterizations of pure-phase BSCF and hybrid BSCF. **a** In situ chemical synthesis of hybrid BSCF material with host phase BSCF, hexagonal-phase BaCl_2_ and cubic-phase SrCl_2_. **b** XRD patterns and refinements. HRTEM images of **c** pure-phase BSCF and **d** hybrid BSCF (scale bar is 2 nm). **e**–**g** EDX spectroscopy line-scan profiles (scale bar is 50 nm).

Here, we design high-valence hybrid perovskite based on the *operando* OER catalytic behaviors aimed at simultaneously achieving ideal or near-ideal ion leaching effects and protecting material crystalline matrix. For proof-of-concept, typical single pervoskite BSCF was first investigated through the introduction of small amounts of soluble hexagonal BaCl_2_ and cubic SrCl_2_ compounds into perovskite structure using a solid-state synthesis method (Fig. 2a). Because of the higher Co valence of raw material used in solid-state synthesis method (Co_3_O_4_) than that in liquid-state synthesis method [Co(NO_3_)_2_], BSCF fabricated under solid-state method tends to show higher Co valence^31^. To optimize the pristine ion combination in hybrid material, a self-assembly synthesis process was conducted. For comparison, pure-phase BSCF was also fabricated via similar synthesis processes. Combined XRD refinements, high-resolution transmission electron microscopy (HRTEM) spectra and energy-dispersive X-ray (EDX) spectroscopy line-scan profiles reveal the structural differences between pure-phase BSCF and hybrid BSCF. XRD refinement results show that pure-phase BSCF and the host phase in hybrid BSCF (79.4 wt.%) both adopt a cubic structure (space group of *Pm*-3*m*) with lattice parameters of *a* = 3.950 and 3.969 Å, respectively, while 11.4 wt.% hexagonal BaCl_2_ (*P*-62*m* space group) and 5.8 wt.% cubic SrCl_2_ (*Fm*-3*m* space group) phases are also contained in hybrid BSCF material (Fig. 2b and Supplementary Table 1). EDX and XRD refinement for the single crystal of host phase in hybrid BSCF were performed to identify its molecular formula as Ba_0.35_Sr_0.65_Co_0.8_Fe_0.2_O_3-*δ*_ (Supplementary Fig. 1). Also, XRD refinement shows that 5.3 wt.% Cl atoms are doped into the host phase of hybrid BSCF. The structural information of pure-phase BSCF and hybrid BSCF was further confirmed by HRTEM images. As shown in Fig. 2c, d, pure-phase BSCF only shows a cubic-phase lattice spacing of 0.28 nm [consistent with (110) plane], while hybrid BSCF nanocomposite exhibits three different lattice distances of 0.28, 0.49, and 0.26 nm corresponding to the (110), (001), and (220) planes of host phase BSCF, hexagonal BaCl_2_, and cubic SrCl_2_, respectively. EDX spectroscopy line-scan spectra also imply the existence of different phases in hybrid BSCF, where the element distribution is inhomogeneous in the hybrid BSCF as compared with the uniformly dispersed elements in pure-phase BSCF (Fig. 2e–g).

Our high-valence BSCF (Co^3+/4+^) samples synthesized from solid-state reaction method are quite different from reported low-valence BSCF5582 materials (Co^2+/3+^) obtained from liquid-state synthesis processes^8,10^, where high-valence Co ions could relieve the effects of electro-derived oxidation for alkaline OER and improve the electrical conductivity of oxides to facilitate electron transport during OER owing to the strong hybridization between Co^4+^ 3*d* orbitals and O 2*p* states^16,36^. Therefore, electronic states of pure-phase BSCF and hybrid BSCF are analyzed here as well. Relative to pure-phase BSCF, the Co *K*-edge XANES spectrum of hybrid BSCF slightly shifts to lower energy (Supplementary Fig. 2a), indicating a relatively lower Co oxidation state in hybrid BSCF^4,8^. This change may attribute to the partial doping of ionic Cl^−^ ions into the host phase of hybrid BSCF^37^. To extract the existing valence configurations in our samples, valence-sensitive soft XAS at the O-*K* edge in fluorescence yield (FY) mode was measured. Generally, the O-*K* pre-edge peaks below 532 eV are related to the unoccupied O 2*p* states hybridized with Co 3*d* state, where the shift of O-*K* pre-edge peaks to lower energies indicates the increase of Co valence^36,38,39^. As shown in Supplementary Fig. 2b, the O-*K* peaks of A and B in our samples are corresponding to Co^3+^ and Co^4+^ ions^39^, respectively, confirming the existence of Co^3+/4+^ ions in both pure and hybrid BSCF materials.

### Electrocatalytic properties and *operando* structural variations

**Fig. 3:**
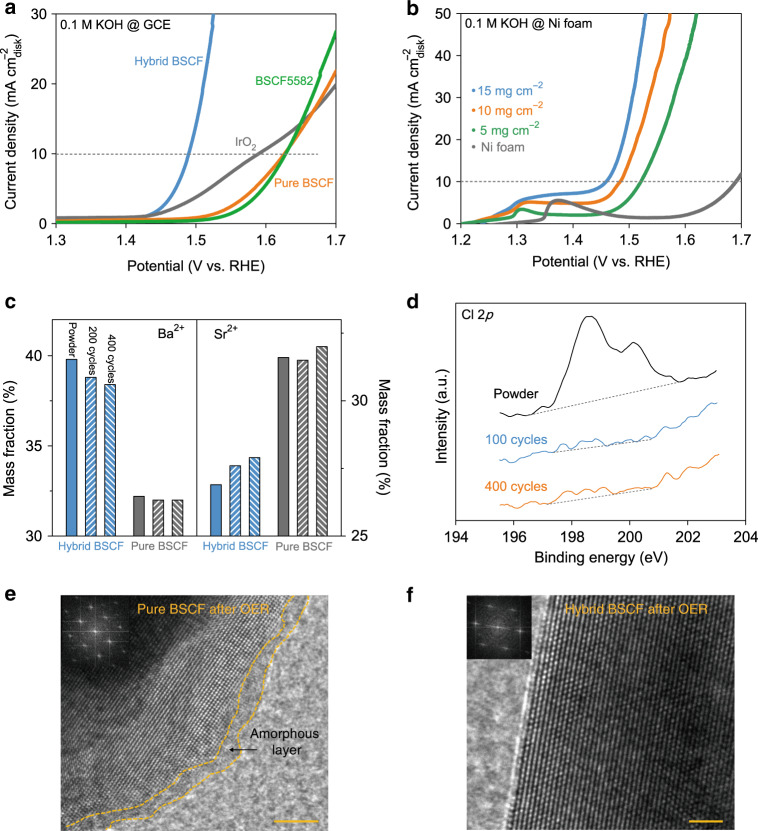
Electrocatalytic OER performance and ion leaching behaviors. **a** OER activities of hybrid BSCF, pure BSCF, BSCF5582, and IrO_2_ catalysts measured on GCE in 0.1 M KOH at 25 °C. **b** OER performance of hybrid BSCF with different catalyst loadings tested on nickel foam. **c** ICP-MS results of Ba^2+^ and Sr^2+^ ions in pristine powders and CV-activated samples. **d** Cl 2*p* XPS spectra of hybrid BSCF before and after CV activations. HRTEM images of **e** pure BSCF after OER (scale bar is 5 nm) and **f** hybrid BSCF after OER (scale bar is 2 nm). Inset figures are the corresponding FFT patters of respective crystalline structures.

As the next step, we investigated the electrocatalytic OER properties of synthesized materials in 0.1 M KOH under ambient conditions and their *operando* structural variations during OER. Traditional OER measurements were conducted following previous reported protocols^16,40^ and all electrode potentials here were *iR*-corrected and referenced to the reversible hydrogen electrode (RHE; Supplementary Fig. 3). Notably, hybrid BSCF shows a robust OER performance on GCE with a low onset overpotential (*η*_1_) of 200 mV (defined as the overpotential at 1 mA cm^−2^_disk_) and a small overpotential of 260 mV required to afford 10 mA cm^−^^2^_disk_ (*η*_10_; Fig. 3a and Supplementary Table 2). Specifically, the *η*_10_ value of 260 mV achieved on hybrid BSCF is about 137 and 100 mV smaller than that of pure-phase BSCF and the benchmark IrO_2_ catalysts, respectively (Fig. 3a and Supplementary Table 2). It is also worth noting that the *η*_10_ value of BSCF5582 (~399 mV, which is similar to previous work^18^) is 139 mV larger than that of hybrid BSCF. Comparing other state-of-the-art cobalt-based perovskites, we found that hybrid BSCF surpasses almost all the candidates for this reaction in 0.1 M KOH reported so far (Supplementary Fig. 4 and Supplementary Table 2). To further improve the OER performance of hybrid BSCF, 3D conductive nickel foam was used as the substrate to offer large electrochemical reaction areas and increase the catalyst loading. As expected, the *η*_10_ value of hybrid BSCF decreases to 230 mV on nickel foam, which is 120 mV lower than that of LaCo_0.8_Fe_0.2_O_3-*δ*_-Ar on nickel foam^41^ (Fig. 3b and Supplementary Table 2). We also normalized the OER current densities to catalyst surface areas measured by Brunauer–Emmett–Teller method (Supplementary Table 3), where hybrid BSCF is still much superior to pure-phase BSCF on GCE (Supplementary Fig. 5a) and the OER activity of hybrid BSCF on nickel foam still enhances with increasing sample loading (Supplementary Fig. 5b). Aside from the remarkable OER activity, hybrid BSCF also exhibits the best OER durability for 100 h among pure/hybrid BSCF and BSCF5582 (Supplementary Fig. 6). To verify the functions of ion leaching effects, we added BaCl_2_ and SrCl_2_ in electrolyte and on electrodes to measure the OER activities of pure BSCF. Expectedly, pure BSCF shows improved OER performance with the functions of BaCl_2_ and SrCl_2_ in liquid and on electrode (Supplementary Fig. 7), confirming the key role of ion leaching effects in the activity differences between hybrid BSCF and pure BSCF. We also measured the OER Faradaic efficiency of hybrid BSCF and confirmed that the side reactions such as chloride evolution reaction cannot occur in 0.1 M KOH solutions theoretically and experimentally (detailed in Supplementary Note 1 and Supplementary Fig. 8). Further investigations of the activity differences will be discussed in the *operando* observations below.

To track the structural variations induced by ion leaching behaviors of our catalysts during OER, electrochemical cycle voltammetry (CV), inductively coupled plasma-mass spectroscopy (ICP-MS), X-ray photoelectron spectroscopy (XPS) as well as *operando* synchrotron XRD were performed. As shown in Supplementary Fig. 9, broad redox pairs appear in CV curves because of the existence of mixed valence states in our perovskites^28^. The maximum current and the redox pairs of CV curves for pure-phase BSCF become smaller during continuous CV scans, while those for hybrid BSCF turn larger (Supplementary Fig. 9), indicating their different structural changes triggered by different ion leaching behaviors. To identify the amounts of leached ions, ICP-MS was conducted on pristine powders and CV-activated samples. We observed that Ba^2+^ ions in pure and hybrid BSCF leach with increasing CV scans and the mass ratio of leached Ba^2+^ ions from hybrid BSCF is much larger than that from pure-phase BSCF (Fig. 3c), implying the pre-leaching of BaCl_2_ and SrCl_2_ from hybrid BSCF. Due to the much larger atomic weight of Ba element relative to Sr element, the whole mass weight of remaining ions in catalysts decreases with Ba leaching, and thus leads to the increased mass fraction of remaining Sr^2+^ ions in our samples (Fig. 3c). We observed Sr^2+^ leaching via XPS spectra, where the peak intensity of Sr 3*d* core-level XPS spectrum becomes lower with CV activation for hybrid BSCF (Supplementary Fig. 10). Also, the leaching of Cl^−^ ions from hybrid BSCF can be verified by Cl 2*p* XPS spectra (Fig. 3d). To go a step closer to the underlying mechanism of structural changes evoked by different ion leaching behaviors in our perovskites during OER conditions, *operando* synchrotron XRD measurements were performed (Supplementary Fig. 11a). As shown in Supplementary Fig. 11b, with increasing OER potentials, the typical XRD peak of cubic perovskite for pure BSCF decreases while that for hybrid BSCF slightly increases, showing that pure BSCF gradually becomes amorphous while hybrid BSCF holds the crystalline structures owing to the pre-leaching of BaCl_2_ and SrCl_2_. HRTEM spectrum of pure BSCF after OER further evidences the formation of amorphous layer (Fig. 3e). We infer that this amorphous layer in pure BSCF would gradually grow and lose into the liquid at the cost of perovskite crystalline matrix, leading to the decrease of XRD peak intensity during OER and its bad OER stability (Supplementary Figs. 11b and 6). Contrarily, because of the pre-leaching of BaCl_2_ and SrCl_2_, the differences of ion concentration between interfacial ions and catalyst ions reduce and thus hybrid BSCF is endowed with abundant time and space to form stable surface structures. As shown in Fig. 3f, hybrid BSCF exhibits stable crystalline structures after OER, where the fast Fourier transformed (FFT) pattern is similar to that of reported edge/face-sharing structures^42–44^. The edge/face-sharing structures are very stable for electrochemical reactions as reported^30,42–44^, which may account for the slight increase of XRD peak intensity and the good OER stability for hybrid BSCF here (Supplementary Fig. 11b and Supplementary Fig. 6). Detailed analysis of edge/face-sharing structures formed during alkaline OER conditions will be discussed below.

### Self-optimized local structures and electronic structures

**Fig. 4:**
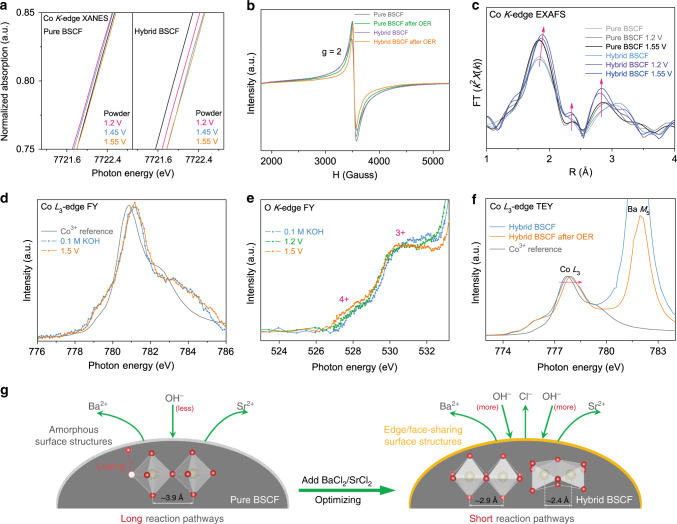
*Operando* spectroscopic characterizations and proposed OER catalytic mechanism. **a** *Operando* Co *K*-edge XANES spectra for pure BSCF and hybrid BSCF. **b** EPR results of samples before OER and after a short-time OER activation. **c** *Operando* Co *K*-edge EXAFS spectra for pure and hybrid BSCF samples (with phase correction). *Operando* soft XAS spectra at **d** Co-*L*_3_ and **e** O-*K* edges in FY mode. **f** Ex situ soft XAS spectra at Co-*L*_3_ and Ba-*M*_5_ edges in TEY mode. **g** Schematic presentations of proposed self-optimizing OER mechanism, where corner-sharing, edge-sharing, and face-sharing Co–Co/Fe metal sites show interatomic distances of 3.9, 2.9, and 2.4 Å, respectively. Single crystal EuCoO_3_ was used as the Co^3+^ standard for Co *L*_3_-edge spectra.

Above, we have studied the crystalline variations evoked by different ion leaching behaviors of our samples for alkaline OER. Here, to further investigate the origin of activity differences between pure BSCF and hybrid BSCF and to reveal the underlying OER catalytic mechanism, *operando* hard and soft XAS characterizations were performed to track the changes of local structures and electronic structures on catalyst surface. It is worth noting that if the OER reaction depth for samples with Co^3+/4+^ is assumed to be ~10 nm^32,45^ then the hard XAS (i.e., XANES and EXAFS) can offer ~58% surface information of our catalysts according to the relationship between the particle size of our sample (~80 nm as shown in Supplementary Fig. 12) and the probing depth of hard X-ray sources (Supplementary Figs. 13 and 14). Relative to hard XAS, soft XAS in FY mode is more sensitive to material surface (~200 nm probing depth)^38^. Therefore, *operando* hard and soft XAS analytical techniques are extremely helpful to glean the changes of local coordinated environments and electronic structures on catalyst surface. With the increase of OER potentials, the *operando* Co-*K* XANES spectrum of pure-phase BSCF first shifts to lower energies and then moves to higher energy positions, showing that its Co valence first decreases and then increases (Fig. 4a). Different from the behaviors of pure BSCF, the Co oxidation state of hybrid BSCF continues to increase under OER conditions (Fig. 4a). According to above observations of surface structural variations, we infer that pure BSCF may sacrifice its lattice oxygen sites and thus exhibit a decrease of Co valence owing to the formation and loss of unstable amorphous surfaces, while the self-optimized edge/face-sharing structures formed on hybrid BSCF endow it with stable lattice oxygen sites for OER processes (Fig. 4g). To further verify this standpoint, oxygen-vacancy-sensitive electron paramagnetic resonance (EPR) was conducted, where the higher peak intensity at *g* = 2 in EPR spectrum demonstrates more oxygen vacancies in materials^46^. EPR results show that the concentration of oxygen vacancies in hybrid BSCF is higher than that in pure BSCF (Fig. 4b) because of the partial doping of ionic Cl^−^ ions into perovskite structure of hybrid BSCF. After a short-time OER treatment, a stronger peak intensity was observed in EPR spectrum of pure-phase BSCF, confirming the increase of oxygen vacancies and the loss of its unstable oxygen sites (Fig. 4b, g). The oxygen vacancies of hybrid BSCF decrease after OER (Fig. 4b). Considering that the adsorption of OH^−^ species is the first step of alkaline OER processes, we thus infer that the decreased oxygen vacancies in hybrid BSCF after OER may be ascribed to the adsorption of OH^−^ species. To verify this standpoint, we analyzed the O 1*s* XPS spectra of hybrid BSCF before OER and after OER. Following standard O 1*s* XPS fitting processes^42,47^, we find that the oxygen vacancies (O_v_) of hybrid BSCF decrease after OER while the amount of adsorbed OH^−^ (O_surf_) becomes larger (Supplementary Fig. 15a, b), confirming the adsorption of OH^−^ on oxygen vacancies.

To further investigate the adsorption ability of OH^−^ species for pure and hybrid BSCF during OER, *operando* Co-*K* EXAFS spectra were analyzed here (detailed EXAFS fitting processes were given in Supplementary Note 2). As shown in Fig. 4c, Supplementary Fig. 16 and Supplementary Table 4, with increasing OER potentials, the peak intensity of the first Co–O coordination shell at ~1.88 Å interatomic distance for pure/hybrid BSCF increases, implying the adsorption of OH^−^ reactants during OER in line with prior findings^4,8^. Besides, compared with pure BSCF, hybrid BSCF exhibits a higher peak intensity of Co–O coordinated shell at the same OER operating potentials (Fig. 4c and Supplementary Table 4). This means that hybrid BSCF indeed shows a better adsorption ability of OH^−^ than pure BSCF (Fig. 4g). In terms of the changes of Co–O bond length, we consider that the average Co–O bond length is comprised of two different types of bonds for oxides during OER: one is Co site coordinated with the lattice oxygen (O^2−^ in Supplementary Fig. 15c); another is coordinated with adsorbed OH^−^ species (namely Co–OH bond in Supplementary Fig. 15c). Owing to the more negative charged state of O^2−^ than OH^−^, the Co–OH bond length is relatively longer than Co-lattice oxygen bond length (Supplementary Fig. 15c). The existence of the long Co–OH bond during OER may compensate the shortened Co-lattice oxygen bond and lead to the nearly unchanged average Co–O bond length for our oxides (detailed in Supplementary Note 3 and Supplementary Fig. 15c), which is also observed in the literatures^4,8,20^.

From the variations of Co–Co/Fe complex shells, we can have insights into the formation of edge/face-sharing structures in our samples during OER^8,18^. Compared with the EXAFS spectra of pristine samples as shown in Fig. 4c, the Co–Co/Fe complex shell at ~2.9 Å interatomic distance for pure/hybrid BSCF appears under OER potentials of 1.2 and 1.55 V, which is the result of the formation of edge-sharing Co–Co/Fe structures under OER conditions^8,18,48^. However, the shell intensity of edge-sharing structures for hybrid BSCF is much larger than that for pure BSCF (Fig. 4c), indicating their different edge-sharing structures. Combining above findings of structural changes for pure and hybrid BSCF (Fig. 3e, f and Supplementary Fig. 11), we can speculate that the edge-sharing structures formed in pure BSCF during OER are amorphous [e.g., oxy(hydroxide)]^8,18^ while those in hybrid BSCF are crystalline structures formed via self-optimized behaviors. Meanwhile, we also observed the shell peak at ~2.41 Å interatomic distance in Fig. 4c for hybrid BSCF under OER conditions, which can be assigned to the face-sharing Co–Co/Fe structures^18^. The formation of stable edge/face-sharing structures in hybrid BSCF via self-optimized behaviors during OER endows it with shorter reaction pathways between Co–Co/Fe metal active sites as compared with pure BSCF to boost OER kinetics and improve OER stability (Fig. 4c, g).

To further track the evolution of electronic structures for hybrid BSCF (e.g., Co/Fe valence states and Co–O covalency), we carried out *operando* soft XAS at the Co-*L*_3_ edge, O-*K* edge in FY mode (Supplementary Fig. 17), XANES at the Fe-*K* edge combined with ex situ soft XAS at the Co-*L*_3_ and Fe-*L*_3_ edges in total electron yield (TEY) mode, where soft XAS in TEY mode is highly sensitive to the material surface information (~5 nm probing depth)^36,38^. Compared with pure Co^3+^ reference in Fig. 4d, the Co-*L*_3_ soft XAS spectrum in FY mode for hybrid BSCF moves to higher energy positions, confirming the existence of Co^3+/4+^ ions in hybrid BSCF obtained from solid-state reaction method^16,31,36^. Moreover, under OER operating conditions, the spectrum weight of Co-*L*_3_ soft XAS spectrum in FY mode for hybrid BSCF slightly shifts to higher energies (Fig. 4d), indicating the increase of Co valence. Ex situ soft XAS spectra at the Co-*L*_3_ edge in TEY mode also show the increase of Co valence on the surface of hybrid BSCF, which can be determined from the shift of Co-*L*_3_ peak to higher energies^49,50^ in Fig. 4f. Meanwhile, the increase of Co valence for hybrid BSCF during OER can be evidenced by soft XAS spectrum at the O-*K* edge in FY mode^39,51^. As shown in Fig. 4e, the O-*K* pre-edge peak intensity of Co^4+^ ions for hybrid BSCF slightly increases with increasing OER potentials, demonstrating the increase of Co^4+^ ions and the enhancement of Co–O covalence^36,38,39^. *Operando* Fe-*K* XANES and ex situ Fe-*L*_2,3_ spectra show that Fe^3+^ ions^52,53^ in hybrid BSCF are stable during OER conditions (Supplementary Fig. 18). The strengthened Co–O covalence and the stable Fe^3+^ ions in hybrid BSCF during OER would couple the active Co/Fe metal active sites and lattice oxygen active sites to trigger possible AEM and LOM processes^16^.

For all above findings, a self-optimizing mechanism on hybrid BSCF evoked by pre-leaching of BaCl_2_ and SrCl_2_ was proposed in Fig. 4g, where hybrid BSCF possesses more advantages in the local structures and electronic structures than pure BSCF for improving the OER activity and stability, including favorable adsorption ability of OH^−^ reactants, stable active lattice oxygen sites and short reaction pathways of Co–Co/Fe metal active sites created by stable edge/face-sharing structures.

### Reliability of strategy and water splitting application

**Fig. 5:**
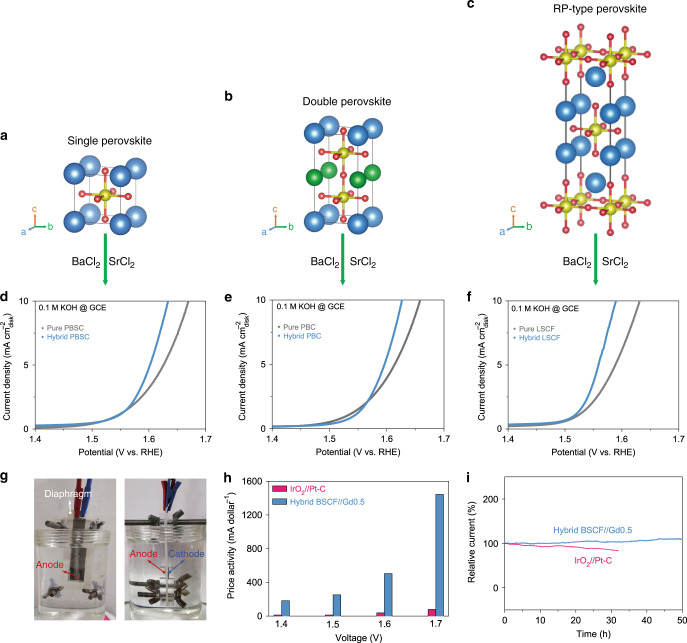
Reliability of strategy and water splitting application. Crystal structures of **a** single, **b** double, and **c** RP-type perovskites. **d**–**f** OER performance of pure and hybrid perovskites measured on GCE in 0.1 M KOH at 25 °C. **g** A back-to-back water electrolyzer. **h** The price activity and **i** stability of hybrid BSCF//Gd0.5 and IrO_2_//Pt-C electrodes for water splitting tested on nickel foam in 1 M KOH at 25 °C. The catalyst loading for water splitting electrodes was 3 mg cm^−2^.

To examine whether our strategy is reliable for other perovskite systems, three perovskites with different crystal structures were prepared and investigated, namely single perovskite Pr_0.5_Ba_0.25_Sr_0.25_CoO_3-*δ*_ (PBSC; Fig. 5a), double perovskite PrBaCo_2_O_6-*δ*_ (PBC; Fig. 5b) and RP-type perovskite La_0.5_Sr_1.5_Co_0.8_Fe_0.2_O_4-*δ*_ (LSCF; Fig. 5c). XRD refinements show that these single, double, and PR-type perovskites exhibit space groups of *Pm*-3*m*, *P*4/*mmm*, and *I*4/*mmm*, respectively (Supplementary Fig. 19 and Supplementary Table 1). Their hybrid perovskites were obtained via mixing respective pure-phase perovskite with soluble BaCl_2_ and SrCl_2_ and then the mixed powders were chemically calcined under high temperature. Notably, all these hybrid perovskites possess higher OER activities than those of their respective pure-phase perovskites, demonstrating the reliability of our strategy (Fig. 5d–f). To move a step closer to the practical application, a back-to-back water electrolyzer was constructed by pairing hybrid BSCF as the anode and BaGd_0.5_La_0.5_Co_2_O_5.75-*δ*_ (Gd0.5)^36^ as the cathode in 1.0 M KOH under the room temperature, where hybrid BSCF and Gd0.5 materials were loaded on nickel foam and separated by a commercial diaphragm (Fig. 5g). In terms of price activity and stability, such perovskite combination is much superior to IrO_2_–Pt/C couple (Fig. 5h, i), showing the competitive potential of our perovskites in alkaline electrolysis applications.

## Discussion

In summary, we apply *operando* electrocatalysis variations (i.e., ion leaching effects) to design promising high-valence hybrid material with self-optimized behaviors, which is different from all the existing design methodologies. Following our design principle, self-assembled high-valence hybrid BSCF nanocomposite shows a low overpotential of 260 mV on GCE for 10 mA cm^−2^ among cobalt-based perovskites for OER in 0.1 M KOH reported so far. Also, this strategy is applicable for other typical single, double, and RP-type perovskites. Combing multiple *operando* spectroscopic techniques (i.e., synchrotron XRD, hard XAS, and soft XAS), we have elucidated the underlying self-optimizing OER mechanism on hybrid BSCF evoked by pre-leaching of BaCl_2_ and SrCl_2_ during OER. Our design principle based on material *operando* changes during electrochemical reactions opens a new avenue for the development of next-generation high-performance electrocatalysts in renewable energy storage and conversion.

## Methods

### Materials synthesis

The hybrid BSCF nanocomposite was prepared by a solid-state reaction method. Starting materials for this synthesis were BaCO_3_ (99.99%, Alba-Aesar), SrCO_3_ (99.99%, Alba-Aesar), SrCl_2_ (99.5%, Alba-Aesar), BaCl_2_ (99.5%, Alba-Aesar), Co_3_O_4_ (99.7%, Alba-Aesar), and Fe_3_O_4_ (99.7%, Alba-Aesar) with a molar ratio of 5.5: 5.5: 0.5: 0.5: 3.33: 0.67. For the first sintering step, a mixture of these raw materials was placed in an Al_2_O_3_ crucible and calcined to 900 °C with a heating rate of 75–90 °C h^−1^ and then kept at 900 °C for 72 h in air. Then the furnace was turned off and the sample was naturally cooled to room temperature. The second and third sintering steps of hybrid BSCF nanocomposite were performed via the same sintering procedure at 1000 and 1100 °C for 72 h after grinding the sintered powders, respectively. The pure BSCF powders were obtained through the similar synthesis processes, where the raw materials of BaCO_3_ (99.99%, Alba-Aesar), SrCO_3_ (99.99%, Alba-Aesar), Co_3_O_4_ (99.7%, Alba-Aesar), and Fe_3_O_4_ (99.7%, Alba-Aesar) were mixed in a molar ratio of 0.35: 0.65: 0.267: 0.067. The first, second, and third sintering steps for pure BSCF were conducted in air at 900 °C for 96 h, 900 °C for 170 h, and 1050 °C for 99 h, respectively. The BSCF5582 sample was fabricated with a nitrate combustion method as described previously^10,18^. In brief, 0.2 M concentration of Ba(NO_3_)_2_, Sr(NO_3_)_2_, Co(NO_3_)_2_∙6H_2_O and Fe(NO_3_)_3_∙9H_2_O (all ˃99%, Aladdin) nitrates were dissolved in deionized water, followed by the addition of 0.1 M glycine (˃99%, Sigma-Aldrich). The mixture was then evaporated at ~90 °C with continuous stirring to obtain a gel. The gel was heated under ~180 °C in an oven until the sparks were observed. The obtained precursor was further calcined in air under 1100 °C for 24 h. The powders of hybrid BSCF, pure BSCF, and BSCF5582 were ball milled for 1 h at a rotation of 400 rpm in a high-energy ball mill (Fritsch Pulverisette 6). The single crystal of the host phase in hybrid BSCF was fabricated via the same molar ratios of raw materials mentioned above (5.5: 5.5: 0.5: 0.5: 3.33: 0.67). The mixed starting materials were first heated at 850 °C for 12 h and then sintered at 1050 °C for 12 h with a heating rate of 1 °C h^−1^. Afterwards, the sample was cooled to 850 °C with a cooling rate of 0.5 °C h^−1^ and then naturally cooled to room temperature. The pure-phase PBSC, PBC, LSCF, and Gd0.5 samples were achieved using a combined ethylenediaminetetraacetic acid-citric acid (EDTA–CA) complexing sol–gel process as detailed in previous works^36,38^. Briefly, stoichiometric amounts of metal nitrates (all ˃99%, Aladdin) were dissolved in deionized water. Then, EDTA and CA were added at a mole ratio of 1:1:2 for total metal ions/EDTA/CA. An NH_3_ aqueous solution was used to complete the complexation and adjust the pH to 6–7. The solutions were heated at ~90 °C under stirring. A gel was obtained and further heated at ~180 °C in air for 5 h to obtain a solid precursor. The solid precursor was further calcined at 1000 °C in air for 24 h. The hybrid PBSC, PBC, and LSCF powders were obtained via mixing respective pure-phase perovskites with BaCl_2_ and SrCl_2_ and then calcinating the mixed powders under 900 °C for 10 h. The Pt/C and IrO_2_ catalysts in this study were purchased from the companies of Johnson Matthey and Macklin, respectively. The commercial diaphragm for water splitting was bought from AGFA Company (Zirfon Perl). The SiC membrane used in *operando* soft XAS experiments was customized from NTT Advanced Technology Corporation.

### Characterizations

XRD data of pure BSCF and hybrid BSCF powders were acquired over a Bruker D8 diffractometer with Cu radiation (*λ* = 1.5418 Å). XRD spectra of other samples in this study were obtained on an X-ray diffractometer of Rigaku Smartlab with filtered Cu-Kα radiation (*λ* = 1.5418 Å). Structural refinements were performed via the TOPAS-4.2 software packages. EDX results of the single crystal were collected on a Phoenix V 5.29 (EDAX) operating at 15 kV. HRTEM and EDX spectroscopy line-scan profiles were achieved using a Tecnai G2 F20 S-TWIN operating at 200 kV. BET and SEM measurements were performed on a Quantachrome Autosorb-iQ3 and a Hitachi S-4800 scanning electron micro-analyzer, respectively. ICP-MS was conducted on a Varian Vista-Pro instrument. XPS spectra were taken through a PHI5000 VersaProbe spectrometer with an Al-Kα X-ray source. EPR spectra were recorded using a Bruker EMX-E EPR spectrometer. Synchrotron XRD, Co *K*-edge XANES and EXAFS, Fe *K*-edge XANES, and soft XAS were performed at the TLS BL01C2 (*λ* = 1.0332 Å), TPS BL44A, TLS BL07A, and TLS BL11A of the NSRRC in Taiwan, respectively. To calibrate the photon energies of Co *K*-edge and Fe *K*-edge spectra, metal Co and Fe foils were used as the references, respectively. CoO, NiO, and Fe_2_O_3_ single crystals were measured simultaneously for the calibration of the energy scales of Co *L*_3_-edge, O *K*-edge, and Fe *L*_2,3_-edge spectra, respectively. The EXAFS data were processed according to the standard procedures using the ATHENA module implemented in the IFEFFIT software packages.

### Electrochemical measurements

Electrochemical OER measurements were conducted on a three-electrode rotating disk electrode configuration (RDE; Pine Research Instrumentation) with a CH Instruments CHI760E potentiostat in O_2_-saturated 0.1 M KOH under room temperature, where GCE (0.196 cm^2^), Ag/AgCl with a double junction, and graphite rod served as the working, reference, and counter electrode, respectively. A rotation rate of 1600 rpm was applied to remove the O_2_ bubbles evolved from the catalyst surface. The working electrode was prepared via dropcasting the homogeneous catalyst ink onto the GCE (5 μL ink). The catalyst ink contains sample powders (10 mg), Super P carbon black (10 mg), absolute ethanol (1 mL), and 5 wt% nafion 117 solution (100 μL). All OER potentials were calibrated to the RHE scale (0.9335 V in 0.1 M KOH), which was obtained using a Pt RDE, a Ag/AgCl, and a graphite rod as the working electrode, reference electrode and counter electrode in H_2_-saturated 0.1 M KOH solutions, respectively. Ohmic losses were corrected by subtracting the ohmic voltage drop from the measured potential using an electrolyte resistance determined from the impedance spectroscopy.

## Supplementary information

Supplementary Information

## Data Availability

All relevant data are available from the corresponding authors on request. The source data underlying Figs. 2b, g, 3a–d, 4a–f, 5d–f, 5h, i and Supplementary Figs. 2–10, 11b, 14b, 15a, b, 16, 18, and 19 are provided as a Source Data file. Source data are provided with this paper.

## Source data

Source Data
