# Supplementary material for: Utilizing ion leaching effects for achieving high oxygen-evolving performance on hybrid nanocomposite with self-optimized behaviors
Source: Nat Commun. 2020 Jul 6;11:3376. doi: 10.1038/s41467-020-17108-5 (PMC7338502; doi:10.1038/s41467-020-17108-5)
Supplement: Supplementary file 1 — Supplementary Information [file 41467_2020_17108_MOESM1_ESM.pdf]

Utilizing ion leaching effects for achieving high oxygen-evolving performance on hybrid nanocomposite with self-optimized behaviors

Guan *et al.*

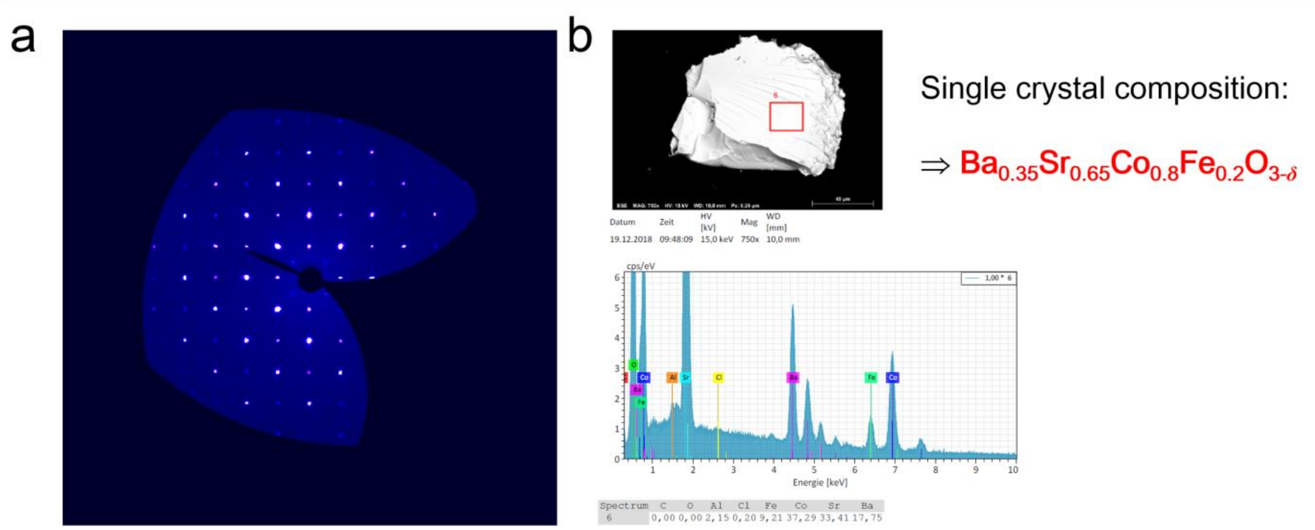

Supplementary Figure 1. Single crystal composition of the host phase in hybrid BSCF. **a** The map for the single crystal of the host phase in hybrid BSCF. **b** EDX results for the single crystal of the host phase in hybrid BSCF. The accurate composition of the host phase was identified from the atom ratios of Ba/Sr and Co/Fe in the EDX table below.

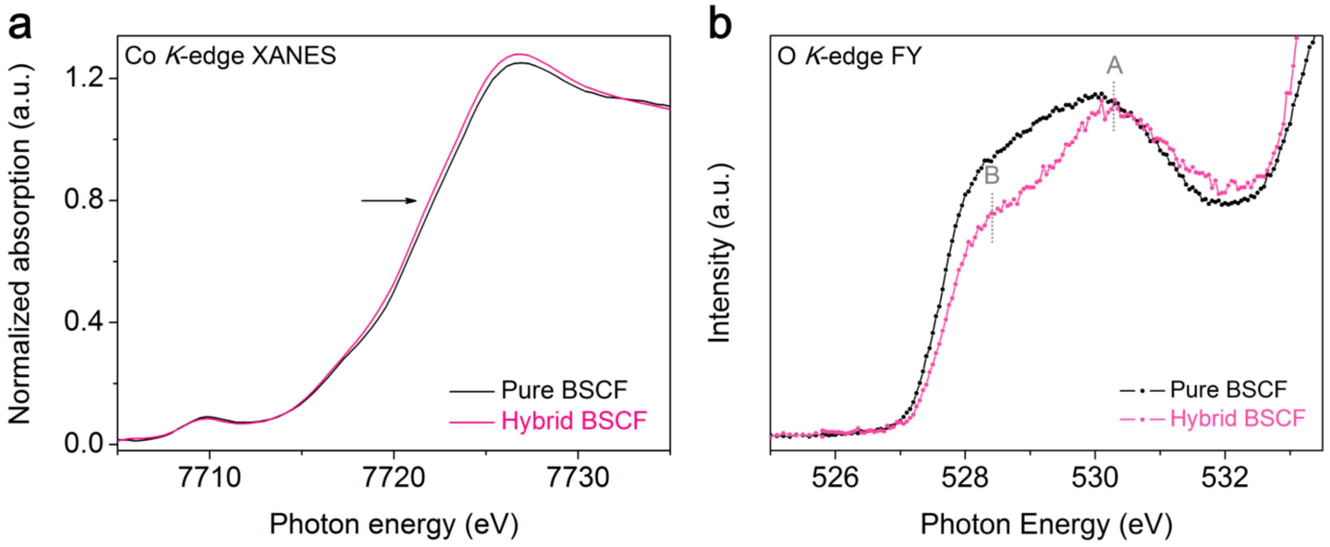

Supplementary Figure 2. Co oxidation states of pure-phase BSCF and hybrid BSCF. **a** Co *K*-edge XANES spectra and **b** soft XAS spectra at the O-*K* edge in FY mode.

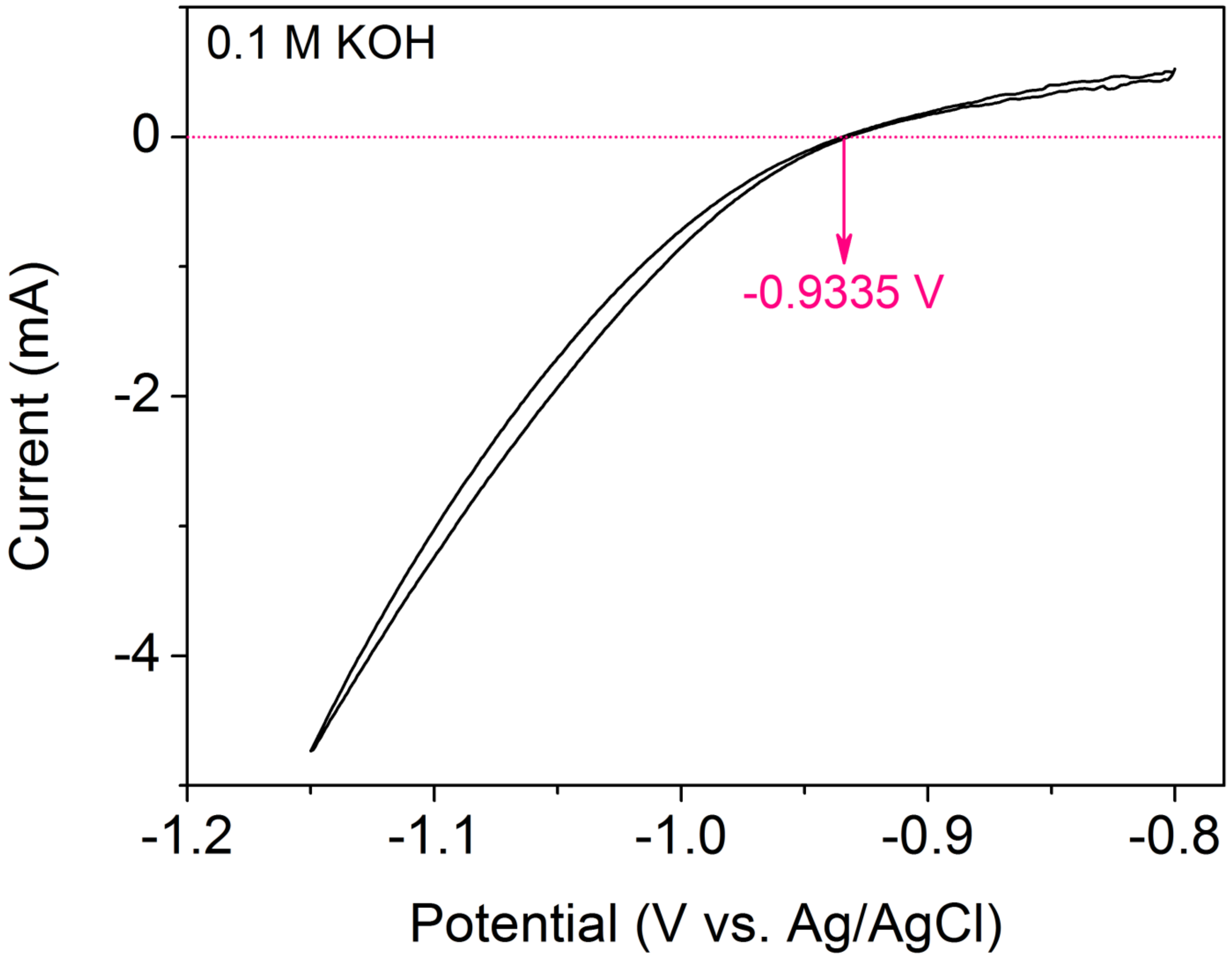

Supplementary Figure 3. RHE calibration of Ag/AgCl reference electrode in 0.1 M KOH under room temperature.

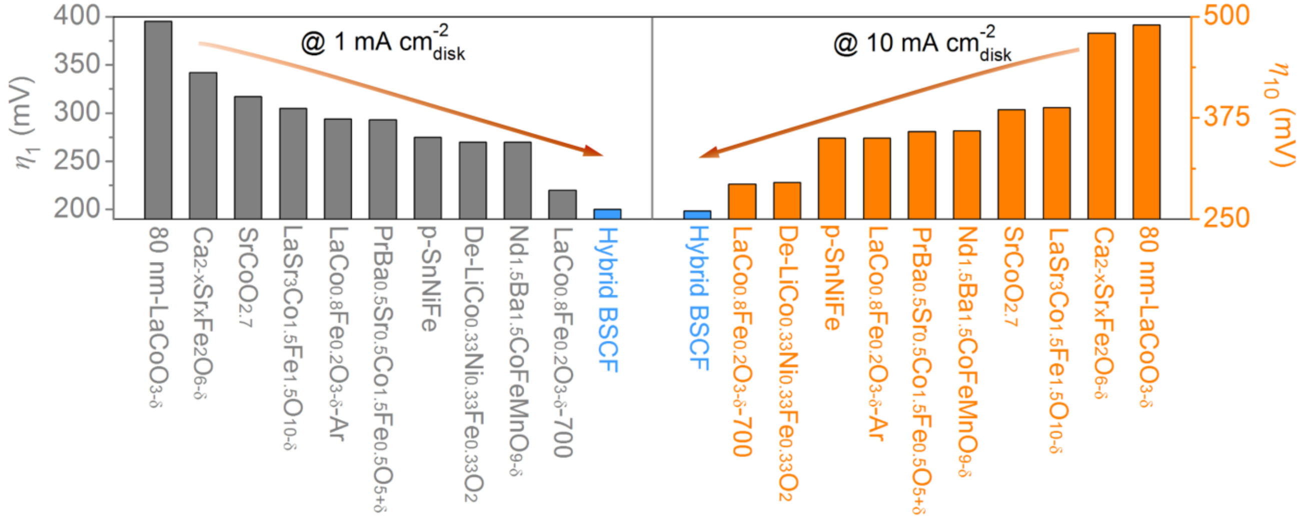

Supplementary Figure 4. OER activity comparisons of overpotentials to afford 1 mA cm^{-2}_{disk} and 10 mA cm^{-2}_{disk} with other reported state-of-the-art cobalt-based perovskites in 0.1 M KOH.

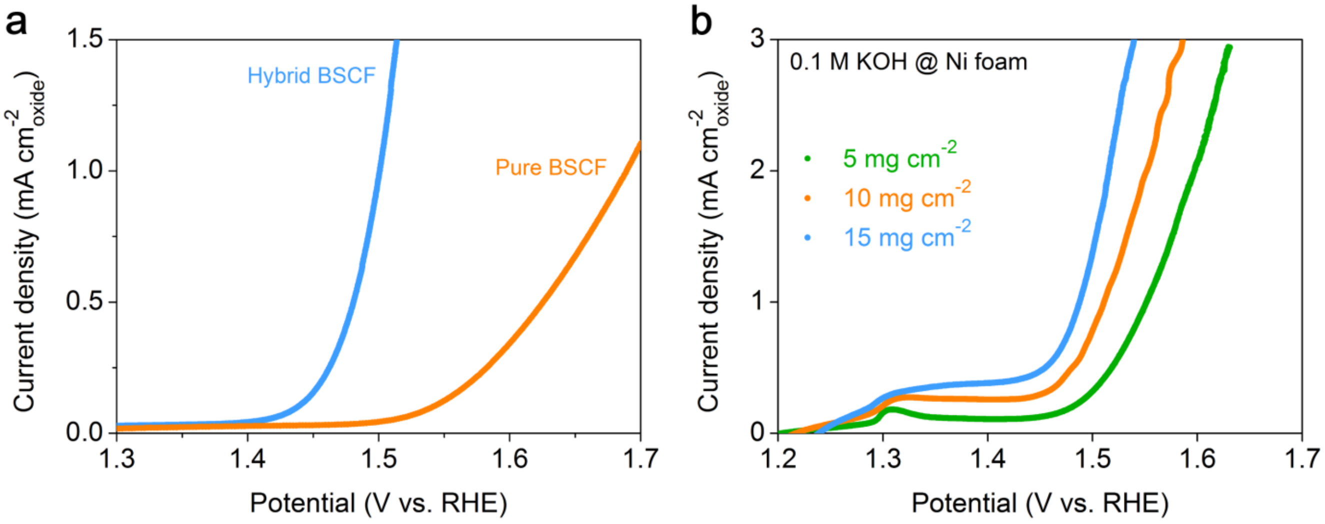

Supplementary Figure 5. Specific OER performance. Specific OER activities **a** on GCE and **b** on Ni foam, where the current densities were normalized to the surface areas of powders.

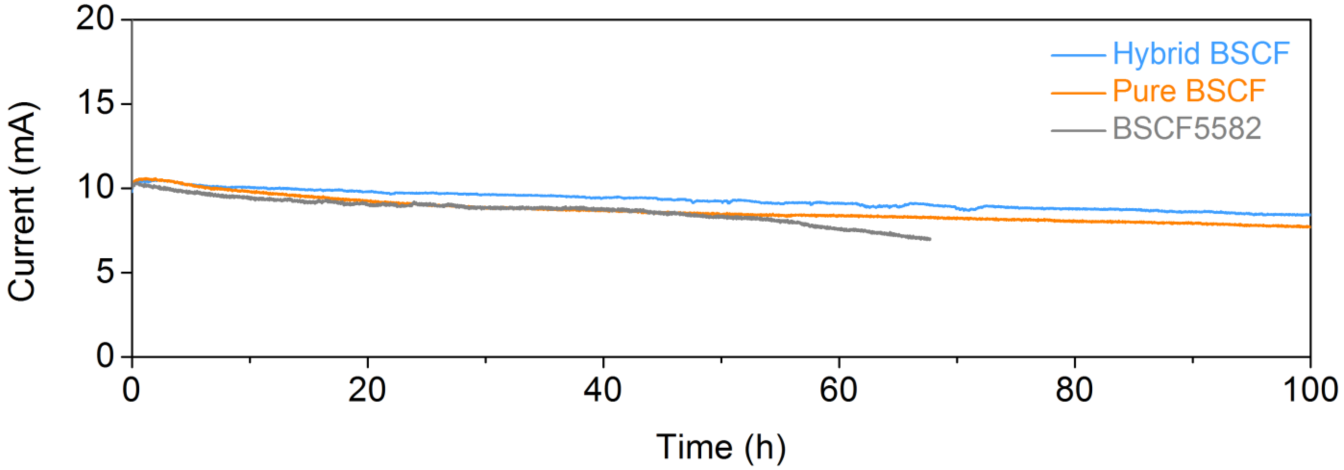

Supplementary Figure 6. Stability of hybrid BSCF, pure BSCF and BSCF5582 loaded on carbon paper with catalyst loading of 3 mg cm^{-2}_{disk} at an initial current of 10 mA. Hg/HgO and graphite rod were used as the reference and counter electrode for the long stability measurements, respectively.

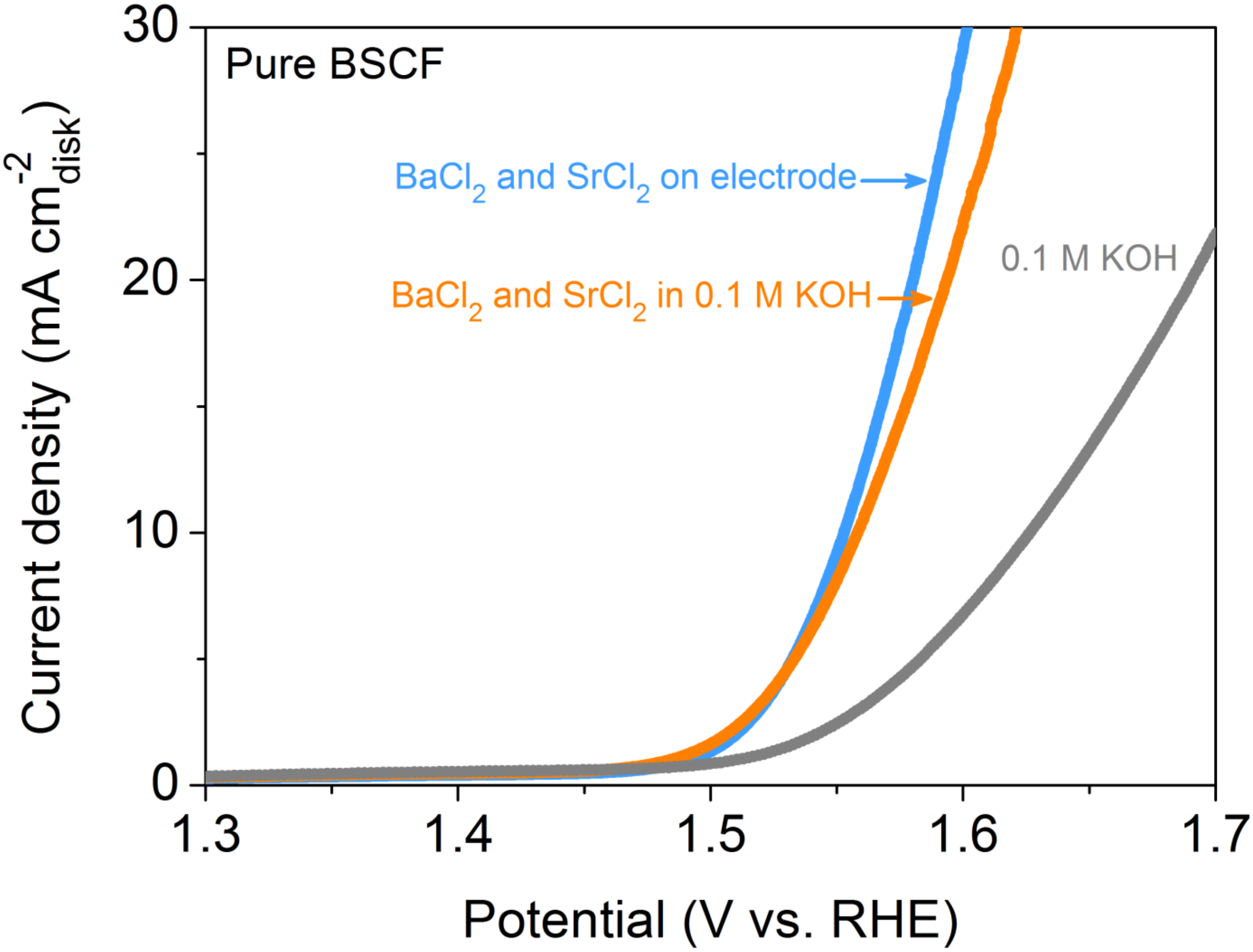

Supplementary Figure 7. Polarization OER curves of pure BSCF in 0.1 M KOH (grey line), pure BSCF in 0.1 M KOH with 0.01 M $BaCl_2$ and 0.01 M $SrCl_2$ (orange line), and pure BSCF with 5 μL $BaCl_2$ and $SrCl_2$ ink on electrode (blue line). The $BaCl_2$/$SrCl_2$ ink contains 10 mg $BaCl_2$, 10 mg $SrCl_2$, 1 mL water, and 0.1 mL 5 wt% Nafion 117 solution.

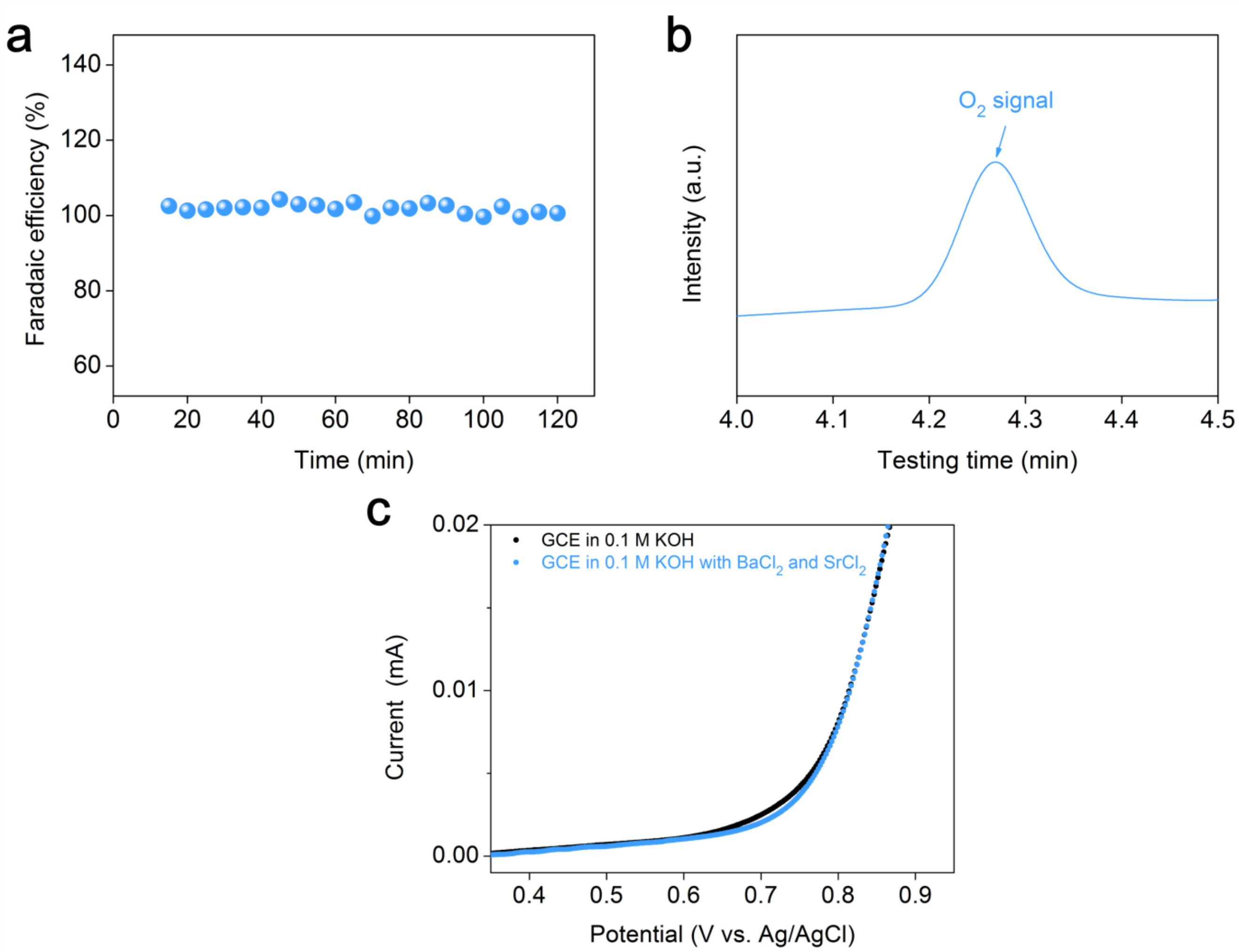

Supplementary Figure 8. OER Faradaic efficiency. **a** OER Faradaic efficiency of hybrid BSCF. **b** Gas chromatography curves in oxygen peak region for hybrid BSCF. **c** OER performance of bare GCE in 0.1 M KOH and 0.1 M KOH with 0.01 M $BaCl_2$ and 0.01 M $SrCl_2$.

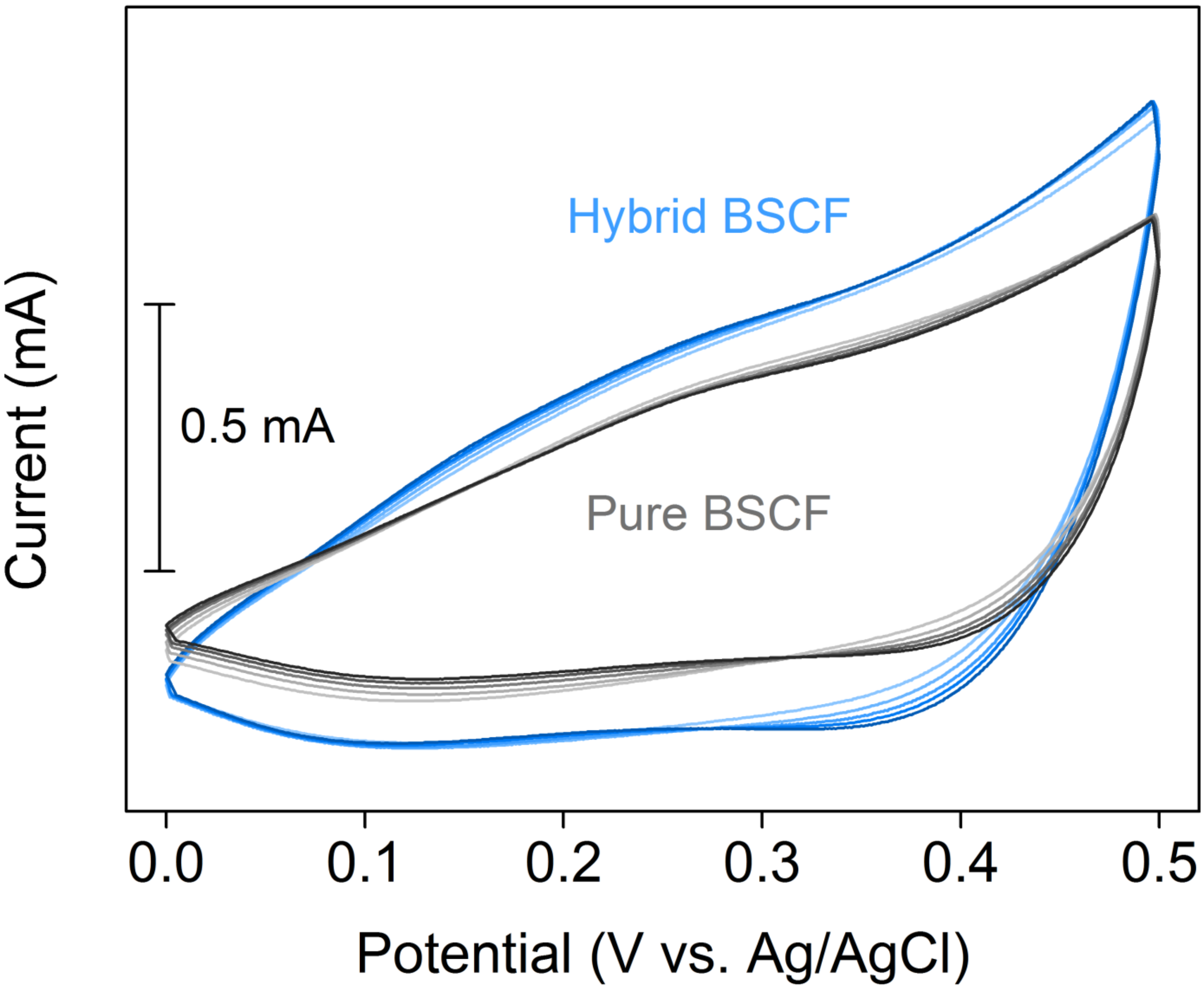

Supplementary Figure 9. CV scans of pure BSCF and hybrid BSCF. The 100th, 200th, 300th, 400th and 500th CV scans were shown in deeper colors.

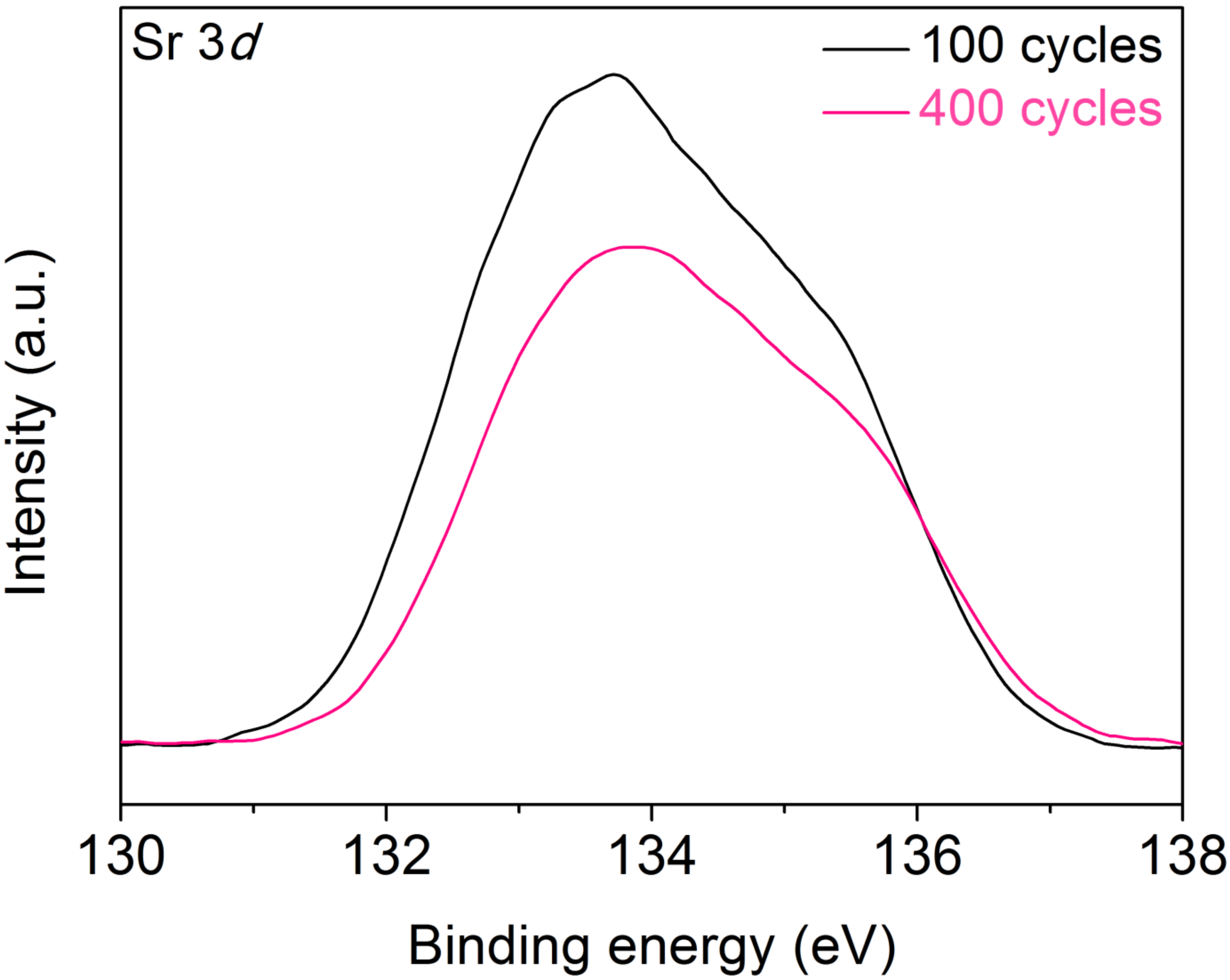

Supplementary Figure 10. Sr 3*d* core-level XPS spectra of hybrid BSCF after 100th and 400th CV cycles.

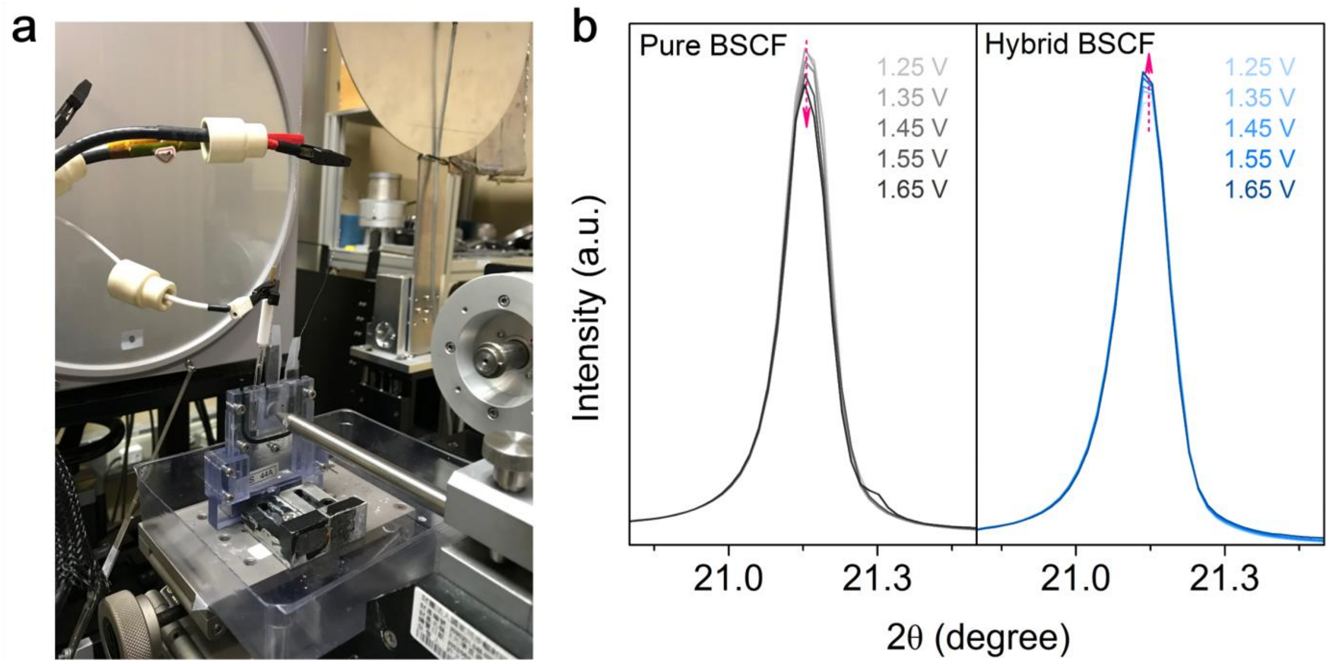

Supplementary Figure 11. *Operando* synchrotron XRD experiments. **a** The setup of *operando* synchrotron XRD experiments. **b** *Operando* synchrotron XRD spectra of pure BSCF and hybrid BSCF under OER conditions.

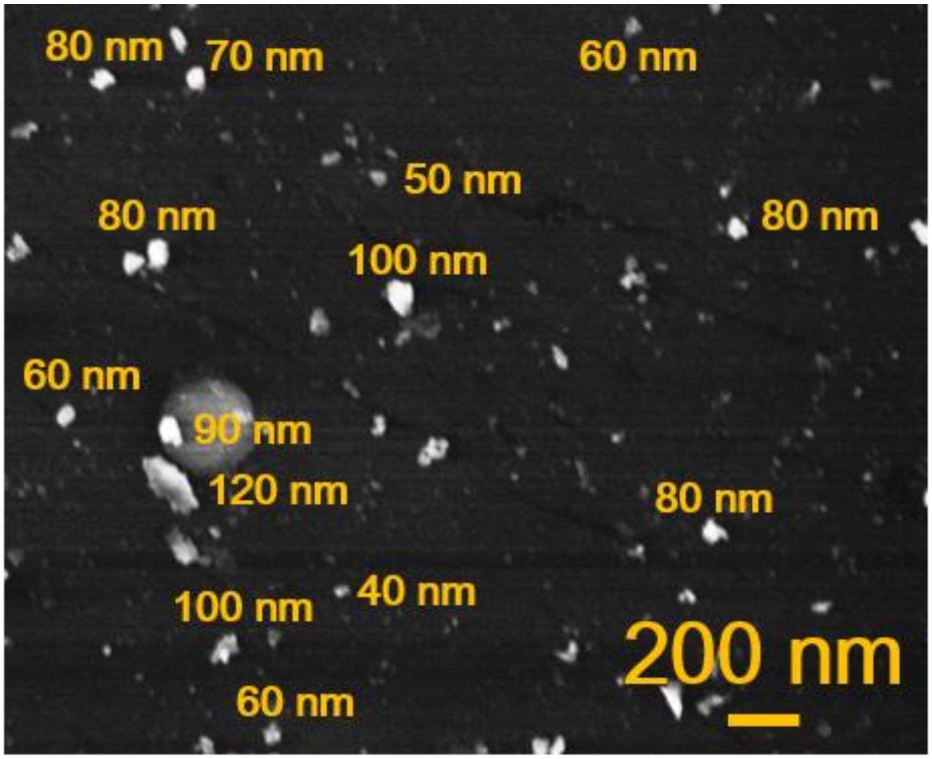

Supplementary Figure 12. SEM image of hybrid BSCF with 200 nm scale bar. The average particle size of hybrid BSCF is ~80 nm.

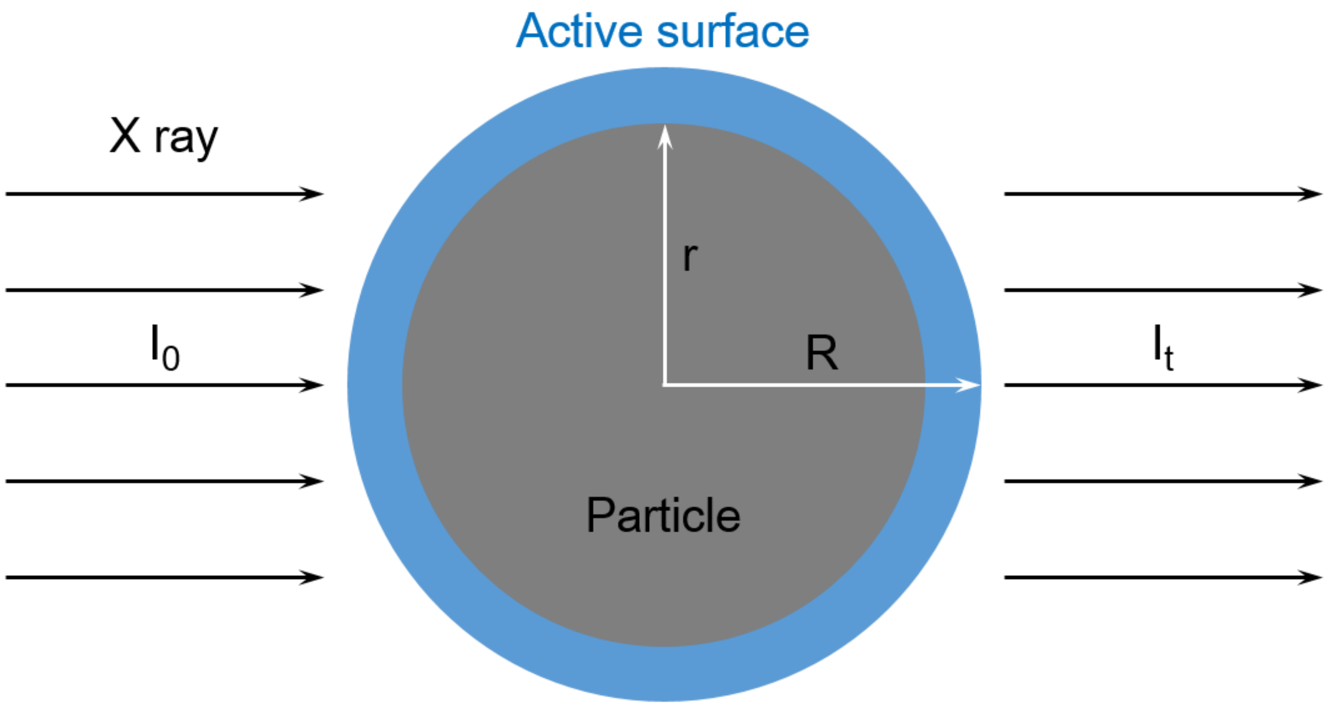

Supplementary Figure 13. The relationships between the particle size of samples and the probing depth of hard X-ray sources. The surface information of catalysts obtained from hard X-ray sources can be calculated as: $\frac{\frac{4}{3}\times\pi\times(R^3-r^3)}{\frac{4}{3}\times\pi\times R^3}$ (where R is the radius of material particle). Assuming that the OER reaction depth for samples with $Co^{3+/4+}$ ions is ~10 nm, then we can calculated the surface information of catalysts obtained from hard X-ray sources is ~58%.

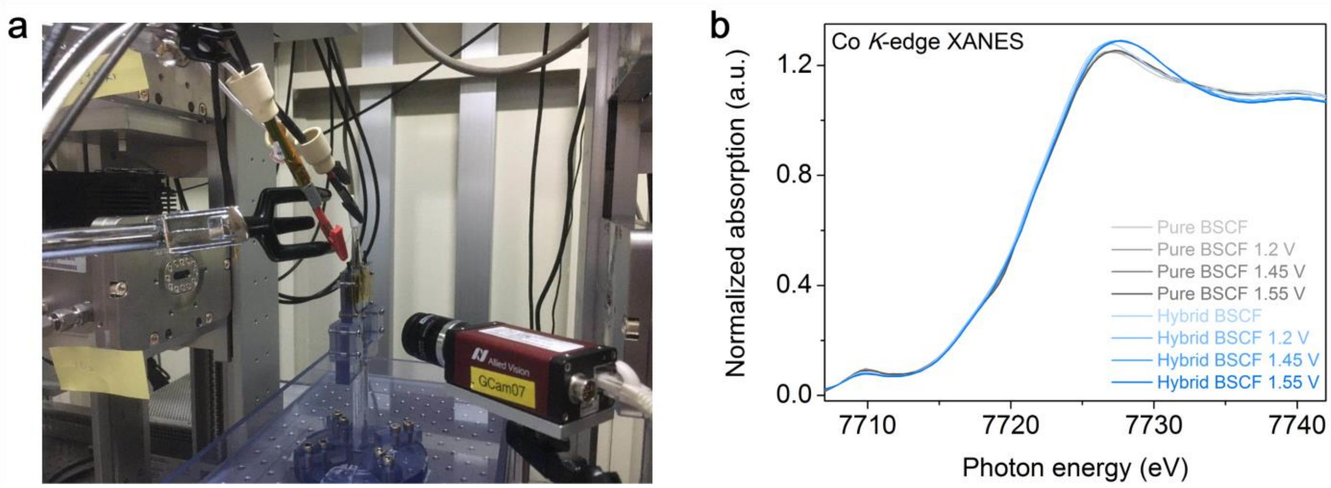

Supplementary Figure 14. *Operando* hard X-ray experiments. **a** The setup of *operando* hard X-ray experiments. **b** *Operando* Co *K*-edge XANES spectra of pure BSCF and hybrid BSCF under OER conditions.

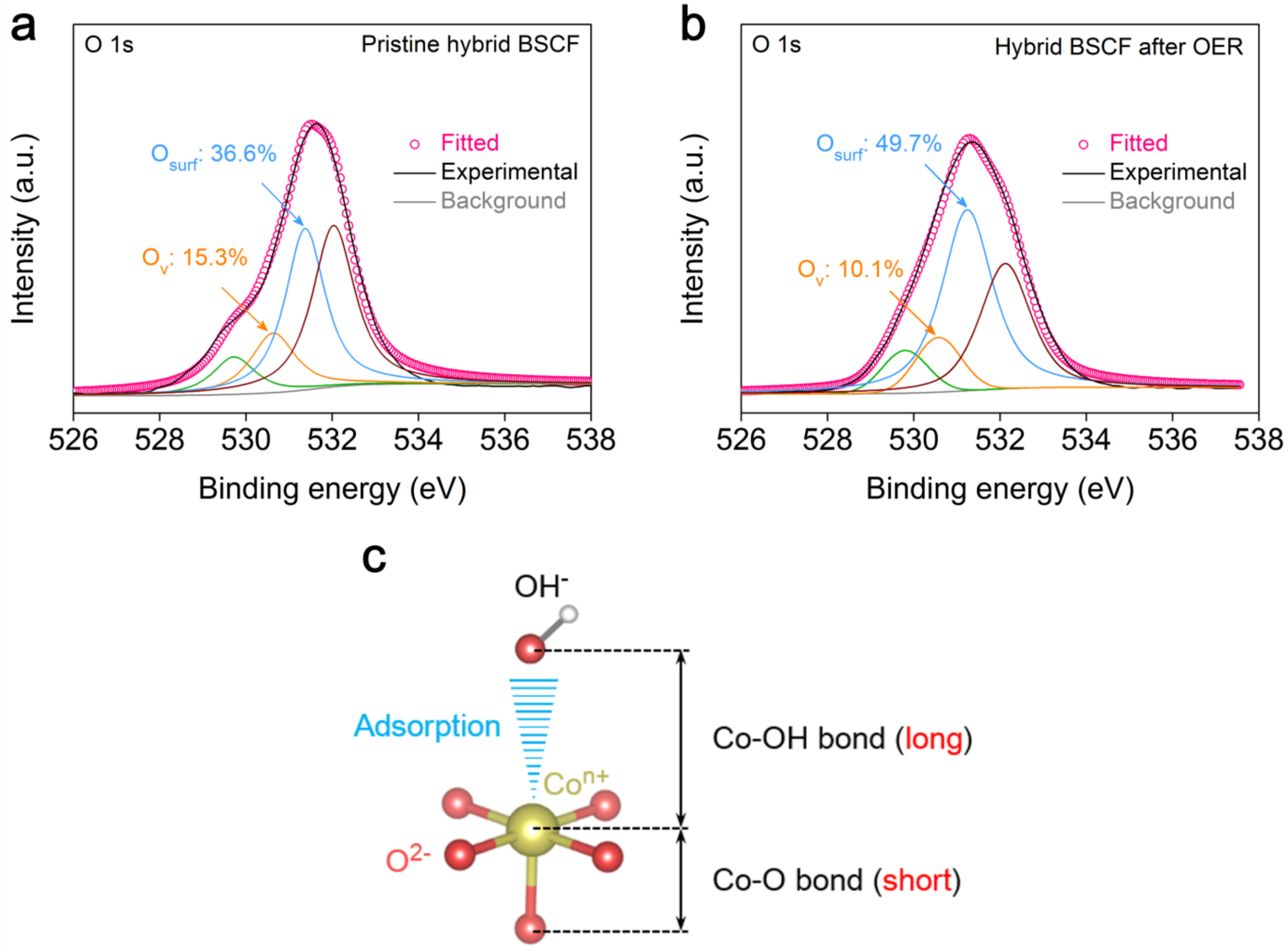

Supplementary Figure 15. The adsorption of OH^- species and Co-O bond variations for alkaline OER. O 1s XPS fitting spectra of **a** pristine hybrid BSCF and **b** hybrid BSCF after OER. **c** Explanation of the variations of Co-O bond length for our samples during OER.

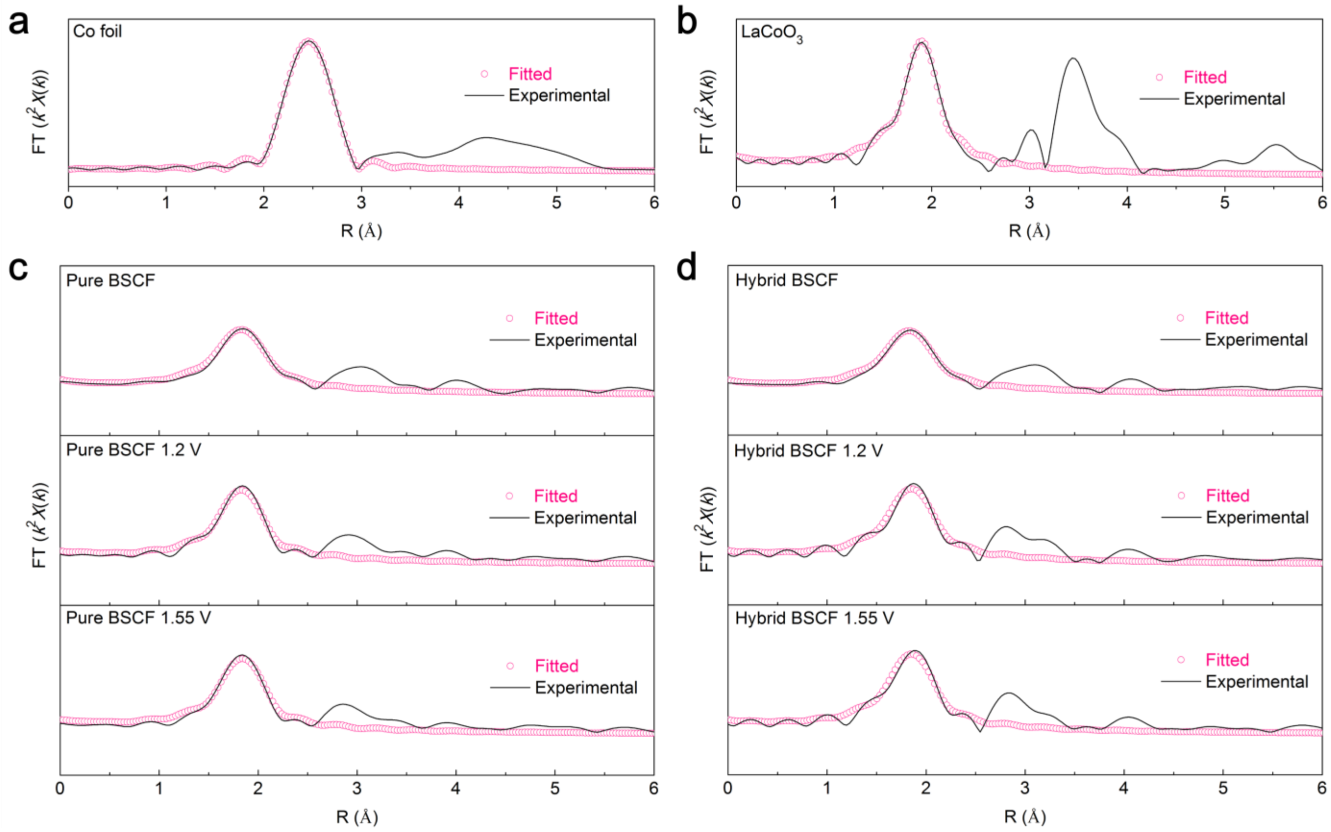

Supplementary Figure 16. Co-*K* edge EXAFS fitting spectra. The k^2-weighted EXAFS fitting spectra (with phase correction) of **a** Co foil, **b** $LaCoO_3$, **c** pure BSCF and **d** hybrid BSCF. The fitting spectra of pristine samples and samples under OER conditions were given in **c** and **d**.

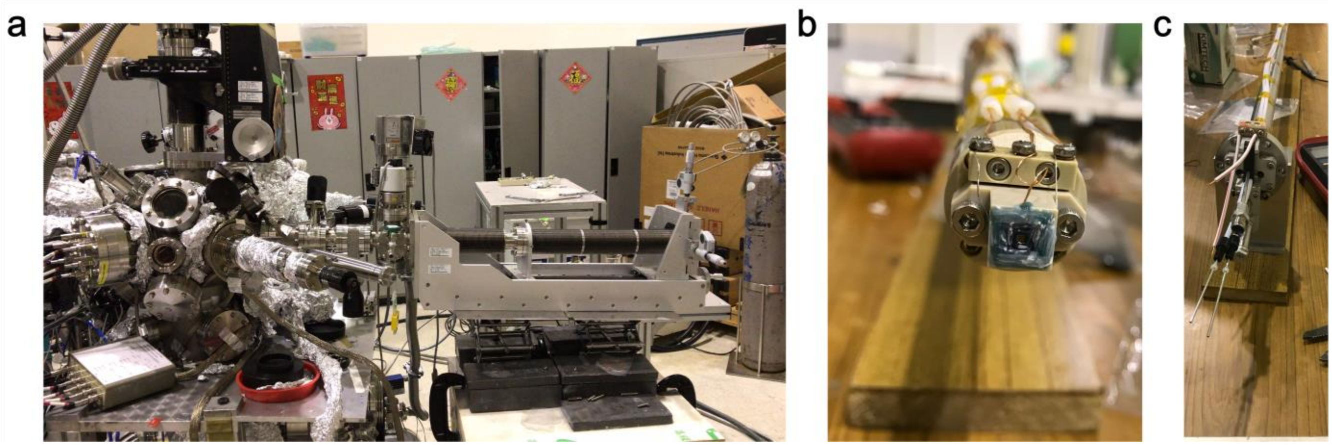

Supplementary Figure 17. The setup of *operando* soft XAS experiments. **a** The whole setup of *operando* soft XAS experiments. **b** The front part of the *operando* electrochemical equipment. **c** The back part of the *operando* electrochemical equipment.

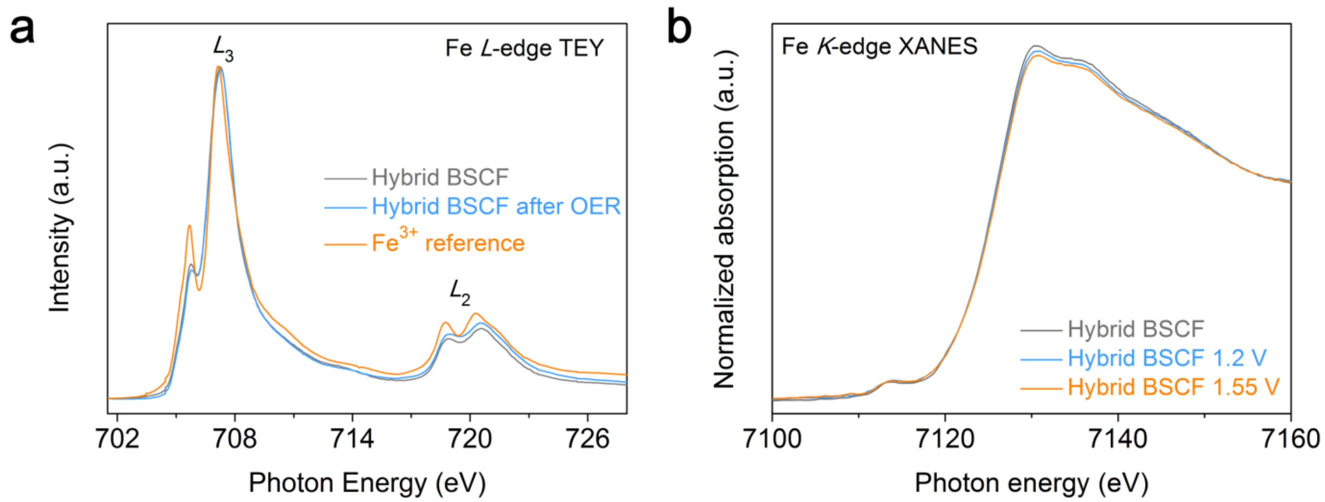

Supplementary Figure 18. Fe valance state of hybrid BSCF. **a** Fe $L_{2,3}$-edge sXAS spectra in TEY mode for hybrid BSCF before OER and after OER, where single crystal Fe_2O_3 was used as the Fe^{3+} standard for Fe $L_{2,3}$-edge spectra. **b** *Operando* Fe *K*-edge XANES spectra of hybrid BSCF under OER conditions.

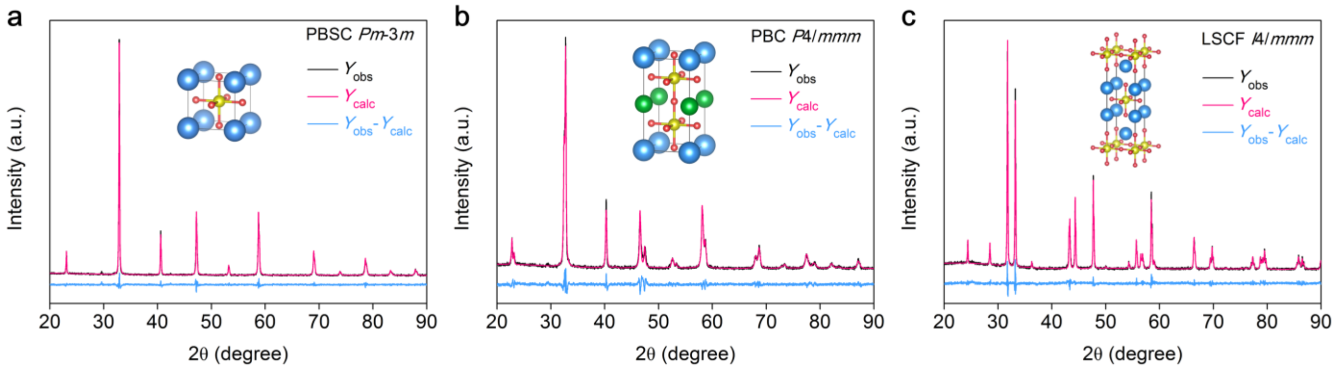

Supplementary Figure 19. XRD refinements. XRD structural refinements of **a** single perovskite $Pr_{0.5}Ba_{0.25}Sr_{0.25}CoO_{3-\delta}$, **b** double perovskite $PrBaCo_2O_{6-\delta}$ and **c** RP-type perovskite $La_{0.5}Sr_{1.5}Co_{0.8}Fe_{0.2}O_{4-\delta}$.

Supplementary Table 1. Results of XRD refinements.

Sample	Phase	Proportion (wt. %)	Space group	a (Å)	b (Å)	c (Å)	GOF
Pure BSCF	BSCF	100	Pm-3m	3.950	3.950	3.950	1.11
	BSCF	79.4	Pm-3m	3.969	3.969	3.969	
Hybrid BSCF	$BaCl_2$	11.4	P-62m	8.051	8.051	4.930	1.18
	$SrCl_2$	5.8	Fm-3m	7.452	7.452	7.452	
	Co_xFe_yO	3.4	R-3cH	5.028	5.028	13.753	
Pure PBSC	PBSC	100	Pm-3m	3.845	3.845	3.845	1.26
Pure PBC	PBC	100	P4/mmm	7.792	7.792	7.662	1.51
Pure LSCF	LSCF	100	I4/mmm	3.809	3.809	12.513	1.31

Supplementary Table 2. Comparisons of OER performance with other state-of-the-art cobalt-based perovskites in 0.1 M KOH.

Catalyst	Electrolyte	Substrate	η_1 (mV)	η_{10} (mV)	Reference
Hybrid BSCF	0.1 M KOH	Glassy carbon	200 mV	260 mV	This work
Hybrid BSCF	0.1 M KOH	Nickel foam	/	230 mV	This work
Pure BSCF	0.1 M KOH	Glassy carbon	281 mV	397 mV	This work
BSCF5582	0.1 M KOH	Glassy carbon	307 mV	399 mV	This work
IrO_2	0.1 M KOH	Glassy carbon	190 mV	360 mV	This work
$LaCo_{0.8}Fe_{0.2}O_{3-\delta}$-700	0.1 M KOH	Glassy carbon	220 mV	293 mV	Ref [1]
De-$LiCo_{0.33}Ni_{0.33}Fe_{0.33}O_2$	0.1 M KOH	Glassy carbon	~270 mV	295 mV	Ref [2]
p-SnNiFe	0.1 M KOH	Glassy carbon	~275 mV	350 mV	Ref [3]
$LaCo_{0.8}Fe_{0.2}O_{3-\delta}$-Ar	0.1 M KOH	Nickel foam	~294 mV	350 mV	Ref [4]
$PrBa_{0.5}Sr_{0.5}Co_{1.5}Fe_{0.5}O_{5+\delta}$	0.1 M KOH	Glassy carbon	~293 mV	358 mV	Ref [5]
$Nd_{1.5}Ba_{1.5}CoFeMnO_{9-\delta}$	0.1 M KOH	Glassy carbon	~270 mV	359 mV	Ref [6]
$SrCoO_{2.7}$	0.1 M KOH	Glassy carbon	~317 mV	~385 mV	Ref [7]
$LaSr_3Co_{1.5}Fe_{1.5}O_{10-\delta}$	0.1 M KOH	Glassy carbon	~305 mV	388 mV	Ref [8]
$Ca_{2-x}Sr_xFe_2O_{6-\delta}$	0.1 M KOH	Glassy carbon	~342 mV	480 mV	Ref [9]
80 nm-$LaCoO_{3-\delta}$	0.1 M KOH	Glassy carbon	~395 mV	490 mV	Ref [10]

Supplementary Table 3. BET results.

Samples	Powders (m^2 g^{-1})
Pure BSCF	8.52 ±0.70
Hybrid BSCF	6.24 ±0.68
Hybird BSCF on Ni electrodes	8.11 ±0.74

All the BET correlation coefficient values are above 99.9%.

Supplementary Table 4. The k^2-weighted EXAFS fitting results.

Samples	Bond type	CN[a]	R (Å)[b]	σ^2 (10^{-3} ×Å^2)[c]	ΔE_0 (eV)[d]	R* factor[e]
Co foil	Co-Co	12	2.49(0.0054)	5.5(0.69)	-4.8(0.9)	0.0065
$LaCoO_3$	Co-O	6.08(0.55)	1.92(0.0083)	3.5(0.97)	-4.0(1.3)	0.0085
Pure BSCF	Co-O	4.14(0.97)	1.87(0.026)	9.1(4.5)	-3.2(2.8)	0.015
Pure BSCF 1.2 V	Co-O	4.17(1.15)	1.88(0.033)	6.3(5.4)	-1.8(3.4)	0.024
Pure BSCF 1.55 V	Co-O	4.19(1.21)	1.89(0.034)	6.2(5.7)	-0.8(3.4)	0.025
Hybrid BSCF	Co-O	4.08(0.96)	1.87(0.027)	9.0(4.4)	-4.0(3.0)	0.013
Hybrid BSCF 1.2 V	Co-O	4.26(1.07)	1.89(0.029)	7.2(4.8)	-2.2(3.1)	0.021
Hybrid BSCF 1.55 V	Co-O	4.42(1.01)	1.89(0.026)	6.9(4.3)	-2.1(2.9)	0.017

The amplitude reduction factor S_0^2 is fixed as 0.722 for all samples, which was obtained by fitting the Co-*K* EXAFS spectrum of Co foil sample.

The k-range (2.7~11) and R-range (1~2.3) for all samples are the same.

[a]CN is coordination number.

[b]R represents interatomic distance (with phase correction).

[c]σ^2 means Debye-Waller factor.

[d]ΔE_0 stands for the difference between the zero kinetic energy value of the sample and that of the theoretical model.

[e]R* factor is a standard function defined by the Standards and Criteria Committee of the International XAFS Society, which weights the quality of fitting.

Supplementary Note 1

OER Faradaic efficiency

We measured the OER Faradaic efficiency of hybrid BSCF and confirmed that the side reactions such as chloride evolution reaction (ClER) cannot occur in 0.1 M KOH solutions (pH = 13) theoretically and experimentally.

First, following traditional measurements of OER Faradaic efficiency by gas chromatography[11], we found that hybrid BSCF shows a ~100% OER Faradaic efficiency [calculated as $\eta = \frac{4F^{*}n_{O2}}{Q}$, where F is the Faraday constant, n_{O2} (mol) is the total amount of produced oxygen (obtained by HOPE GC-9860 gas chromatography) and Q (C) is the total charge passed through the cell] as shown in **Supplementary Fig. 8a, b**, ruling out the existence of side reactions.

Furthermore, we also tested the OER performance of GCE without samples in 0.1 M KOH and 0.1 M KOH with $BaCl_2$ and $SrCl_2$, where the current and potential are almost the same in 0.1 M KOH and 0.1 M KOH with $BaCl_2$ and $SrCl_2$ (**Supplementary Fig. 8c**), verifying that no side reactions would occur to affect the measured currents and potentials.

Thermodynamically, it is well known that only OER is possible under OER potentials in high pH solutions (especially at pH = 13) and ClER only occurs in acidic solutions as studied in the thermodynamic Pourbaix diagrams in the work of Tong *et al.*[12].

Therefore, theoretically and experimentally, we have confirmed that no side reactions occurred in our measured system (0.1 M KOH).

Supplementary Note 2

Co-*K* EXAFS fitting processes

First, we fitted the k^2-weighted EXAFS spectrum of 12-coordinated Co foil and obtained the $S0^2$ value of 0.722 (**Supplementary Table 4**). Then, the $S0^2$ value is fixed for the k^2-weighted EXAFS spectrum of single crystal $LaCoO_3$. We obtained the Co-O coordination number of ~6 (namely no oxygen vacancies) and Co-O bond length of 1.92 Å for $LaCoO_3$ (**Supplementary Table 4**), which are in agreement with previous reported results[13]. As the next step, we fixed the $S0^2$ value for the k^2-weighted EXAFS spectra of pristine pure/hybrid BSCF and samples under OER conditions to obtain the Co-O coordination number and Co-O bond length. Owing to the existence of oxygen vacancies in our samples known from the EPR results in **Fig. 4b**, the Co-O coordination numbers of our samples (4.1~4.4) are smaller than that of single crystal $LaCoO_3$ as shown in **Supplementary Table 4**, which are well consistent with the reported EXAFS fitting results of oxides with oxygen vacancies[13,14]. Besides, the Co-O bond length of our samples (~1.88 Å) is shorter than that of pure Co^{3+} reference $LaCoO_3$ (1.92 Å), confirming that the Co valence of our samples is higher than Co^{3+}. The k^2-weighted EXAFS fitting spectra are shown in **Supplementary Fig. 16**.

Supplementary Note 3

Explanations of the changes of Co-O bond length during OER

In terms of the changes of Co-O bond length, we consider that the average Co-O bond length is comprised of two different types of bonds for oxides during OER: one is Co site coordinated with the lattice oxygen (O^{2-} in **Supplementary Fig. 15c**); another is coordinated with adsorbed OH^- species (namely Co-OH bond in **Supplementary Fig. 15c**). In general, Co-O bond length should shorten with increasing Co valence for oxides without adsorption of other oxygen-containing species.

However, we have verified the adsorption of OH^- species on our oxides during OER from *operando* EXAFS results in **Supplementary Table 4**, where the increased Co-O coordination number of our samples during OER is ascribed to the adsorption of OH^- species[1,14,15]. To further confirm the adsorption of OH^- species, we analyzed the O 1s XPS spectra of hybrid BSCF before OER and after OER. Following standard O 1s XPS fitting processes[16,17], we find that the oxygen vacancies (O_v) of hybrid BSCF decrease after OER while the amount of adsorbed OH^- (O_{surf}) becomes larger (**Supplementary Fig. 15a**, **b**), verifying the adsorption of OH^- on oxygen vacancies and leading to the increased Co-O coordination number of our samples.

Owing to the more negative charged state of O^{2-} than OH^-, the connection between O^{2-} and Co cations should be stronger than that between OH^- and unsaturated Co cations, leading to the relatively longer Co-OH bond length than Co-O bond length (**Supplementary Fig.**

15c). From Co-K EXAFS results (**Supplementary Table 4**), we can observe that the Co-O coordination number increases with increasing OER potentials, namely the amount of adsorbed OH^- species becomes larger during OER[1,14,15]. Therefore, although the bond length of Co sites coordinated with lattice oxygen sites becomes shorter due to the effects of electro-derived oxidation OER processes, the existence of the long Co-OH bond may compensate the shortened Co-lattice oxygen bond and lead to the nearly unchanged average Co-O bond length for our oxides. Such observations have been evidenced by many other reported studies as well[1,14,15], where the average Co-O bond length did not shorten with increasing Co valence.

Supplementary References

1 Song, S. et al. *Operando* X-ray spectroscopic tracking of self-reconstruction for anchored nanoparticles as high-performance electrocatalysts towards oxygen evolution. *Energy Environ. Sci.* **11**, 2945-2953 (2018).

2 Lu, Z. et al. Electrochemical tuning of layered lithium transition metal oxides for improvement of oxygen evolution reaction. *Nat. Commun.* **5**, 4345 (2014).

3 Li, B. et al. Regulating p-block metals in perovskite nanodots for efficient electrocatalytic water oxidation. *Nat. Commun.* **8**, 934 (2017).

4 Li, B., Tang, C., Wang, H., Zhu, X. & Zhang, Q. An aqueous preoxidation method for monolithic perovskite electrocatalysts with enhanced water oxidation performance. *Sci. Adv.* **2**, e1600495 (2016).

5 Zhao, B. et al. A tailored double perovskite nanofiber catalyst enables ultrafast oxygen evolution. *Nat. Commun.* **8**, 14586 (2017).

6 Kim, N. I. et al. Oxygen-deficient triple perovskites as highly active and durable bifunctional electrocatalysts for oxygen electrode reactions. *Sci. Adv.* **4**, eaap9360 (2018).

7 Mefford, J. T. et al. Water electrolysis on $La_{1-x}Sr_xCoO_{3-\delta}$ perovskite electrocatalysts. *Nat. Commun.* **7**, 11053 (2016).

8 Liu, S., Luo, H., Li, Y., Liu, Q. & Luo, J. L. Structure-engineered electrocatalyst enables highly active and stable oxygen evolution reaction over layered perovskite

$LaSr_3Co_{1.5}Fe_{1.5}O_{10-\delta}$. *Nano Energy* **40**, 115-121 (2017).

9 Hona, R. K. & Ramezanipour, F. Remarkable oxygen-evolution activity of a perovskite oxide from the $Ca_{2-x}Sr_xFe_2O_{6-\delta}$ series. *Angew. Chem. Int. Ed.* **58**, 2060-2063 (2019).

10 Zhou, S. et al. Engineering electrocatalytic activity in nanosized perovskite cobaltite through surface spin-state transition. *Nat. Commun.* **7**, 11510 (2016).

11 Zhang, B. et al. Homogeneously dispersed multimetal oxygen-evolving catalysts. *Science* **352**, 333-337 (2016).

12 Tong, W. et al. Electrolysis of low-grade and saline surface water. *Nat. Energy* **1-11**, doi:10.1038/s41560-020-0550-8 (2020).

13 Chen, Y. et al. Exceptionally active iridium evolved from a pseudo-cubic perovskite for oxygen evolution in acid. *Nat. Commun.* **10**, 572 (2019).

14 Fabbri, E. et al. Dynamic surface self-reconstruction is the key of highly active perovskite nano-electrocatalysts for water splitting. *Nat. Mater.* **16**, 925-931 (2017).

15 Kim, B. J. et al. Functional role of Fe-doping in Co-based perovskite oxide catalysts for oxygen evolution reaction. *J. Am. Chem. Soc.* **141**, 5231-5240 (2019).

16 Zhu, Y. et al. A high-performance electrocatalyst for oxygen evolution reaction: $LiCo_{0.8}Fe_{0.2}O_2$. *Adv. Mater.* **27**, 7150-7155 (2015).

17 Xu, X. et al. Earth-abundant silicon for facilitating water oxidation over iron-based perovskite electrocatalyst. *Adv. Mater. Interfaces* **5**, 1701693 (2018).
